# Moment Estimation in Paired Comparison Models with a Growing Number of Subjects

**DOI:** 10.3390/e28030314

**Published:** 2026-03-11

**Authors:** Qiuping Wang, Lu Pan, Ting Yan

**Affiliations:** 1 School of Mathematics and Statistics, Zhaoqing University, Zhaoqing 526000, China; 2 School of Mathematics and Statistics, Shangqiu Normal University, Shangqiu 476000, China; panlulu@mails.ccnu.edu.cn; 3 Department of Statistics, Central China Normal University, Wuhan 430079, China; tingyanty@mail.ccnu.edu.cn

**Keywords:** consistency, central limit theorem, method of moments, paired comparison, parameter estimation, sparse

## Abstract

When the number of subjects, *n*, is large, paired comparisons are often sparse. Here, we study statistical inference in a class of paired comparison models parameterized by a set of merit parameters, under an Erdös–Rényi comparison graph, where the sparsity is measured by a probability $p_{n}$ tending to zero. We use the moment estimation base on the scores of subjects to infer the merit parameters. We establish a unified theoretical framework in which the uniform consistency and asymptotic normality of the moment estimator hold as the number of subjects goes to infinity. A key idea for the proof of the consistency is that we obtain the convergence rate of the Newton iterative sequence for solving the estimator. We use the Thurstone model to illustrate the unified theoretical results. Further extensions to a fixed sparse comparison graph are also provided. Numerical studies and real data analysis illustrate our theoretical findings.

## 1. Introduction

Subjects are repeatedly compared in pairs in a wide spectrum of situations, including sports games [1], ranking of scientific journals [2,3], the quality of product brands [4] and crowdsourcing [5]. For instance, one team plays with another team in basketball; papers in one journal cite papers in another journal; one consumer chooses one product over another; workers in a crowdsourcing setup are asked to compare pairs of items.

One of the fundamental problems in paired comparison analysis is to derive a fair and reliable ranking of all subjects based on observed comparison data. In round-robin tournaments—where every pair of subjects competes sufficiently many times—a natural ranking can be directly obtained from the number of wins, as the full pairwise comparisons eliminate biases from incomplete matchups. However, in most practical scenarios (e.g., sports leagues, crowdsourcing evaluations, or journal rankings), comparisons are often sparse (not all pairs interact) and stochastic (outcomes contain random noise), leading to unreliable direct rankings based solely on raw win counts. To address this issue, paired comparison models have been developed to statistically infer the underlying merit parameters of subjects and generate objective rankings; see the classic monograph by [6] for a comprehensive overview of such models and their theoretical foundations. Statistical models not only provide a method of ranking all subjects but are also tools for making inferences on the merits of subjects (e.g., testing whether two subjects have the same merit).

Here, we are concerned with a class of paired comparison models that assign one merit parameter to each subject and assume that the win–loss probability of any pair only depends on the difference between their merit parameters. Specifically, the probability of subject *i* winning *j* is

(1) $$P\left(i\;\mathrm{wins}\;j\right)=F\left(\beta_{i}-\beta_{j}\right),\;\;i,j=0,1,\ldots ,n;i\neq j,$$

where *F* is a known cumulative distribution function satisfying F(x)=1−F(−x), $\beta_{i}$ is the merit parameter of subject *i* and n+1 is the total number of subjects. The well-known Bradley–Terry model [7], which dates back to at least 1929 [8], and the Thurstone model [9], are two special cases of Model (1). The former postulates the logistic distribution of F(x), while the latter postulates the normal distribution.

In the standard setting that *n* is fixed and the number of comparisons in each pair goes to infinity, the theoretical properties of Model (1) have been widely investigated in Chapter 4 of [6]. In the opposite scenario that *n* goes to infinity and each pair has a fixed number of comparisons, ref. [10] proved the uniform consistency and asymptotic normality of the maximum likelihood estimator (MLE) in the Bradley–Terry model.

When the number of subjects is large, paired comparisons are often sparse. Taking the NCAA Division I FBS (Football Bowl Subdivision) regular season, for example, a team plays with at most 14 other teams among a total of 120 teams. The observed comparisons can be represented in a comparison graph with n+1 nodes denoting subjects and a weighted edge between two nodes denoting the number of comparisons. The Erdös–Rényi comparison graph has been widely considered in the literature, e.g., [1,11,12,13], where the number of comparisons between any two subjects follows a binomial distribution $\left(T,p_{n}\right)$, and $p_{n}$ measures the sparsity. Under a very weak condition on the sparsity on $p_{n}$, ref. [13] established the uniform consistency and asymptotic normality of the MLE in the Bradley–Terry model by extending the proof strategies in [10].

Moreover, ref. [14] considered a fixed sparse comparison graph by controlling the length from one subject to another subject with 2 or 3, in which the consistency and asymptotic normality of the MLE also hold. Inference in the high-dimensional setting under the Bradley–Terry model and some generalized versions has also attracted great interest in the machine learning literature; the upper bounds of various errors are established under different conditions [e.g., the $\ell_{1}$ error $∥\hat{\beta }{-\beta ∥}_{1}$ in [15,16], the mean square error in [17], the bias $E∥\hat{\beta }{-\beta ∥}_{\infty }$ in [18]. Under the assumption that the log-likelihood function is strictly convex, ref. [1] establish the uniform consistency of the MLE in general paired comparison models. However, the asymptotic theory of moment estimation under sparse paired comparison models remains largely underdeveloped in the literature. Existing theoretical developments focus almost exclusively on maximum likelihood estimation (MLE), which relies on strict distributional assumptions and is computationally demanding in high-dimensional settings. In contrast, this paper develops the method of moments (MOM), which avoids full distribution assumptions and maintains computational simplicity while achieving comparable asymptotic properties. The primary novelty of this work is to extend the asymptotic theory of high-dimensional sparse paired comparison models from MLE to the method of moments, establishing a parallel and complementary theoretical framework.

We further elaborate on the advantages of the method of moments (MOM) for high-dimensional sparse paired comparison models. Beyond computational efficiency, MOM has two key strengths over maximum likelihood estimation (MLE) for practical inference: (1) Robustness—the moment estimator is much less sensitive to outliers (e.g., upsets in our NFL data) in sparse settings, where MLE can be biased by extreme observations; (2) Quasi-likelihood compatibility—MOM relies only on moment conditions and avoids strict distributional assumptions, making it robust to model misspecification in high-dimensional sparse scenarios. These merits justify the use of MOM in this study, and our subsequent analysis establishes its asymptotic properties as the core theoretical contribution.

The main contributions of this paper are as follows. First, we develop the moment estimation, instead of the maximum likelihood estimation (MLE) or Bayesian estimation, based on the scores of subjects (i.e., the number of wins) to estimate the merit parameters in Model (1). The reason why we prioritize moment estimation over MLE is that it is natural to rank subjects according to their scores and the computation based on moment equations is simpler, especially in high-dimensional sparse settings where MLE may suffer from numerical instability due to nonlinear optimization. When F(·) belongs to the exponential family distribution, both estimations are identical. Second, under an Erdös–Rényi comparison graph, we establish a unified theoretical framework in which the uniform consistency and asymptotic normality of the moment estimator hold when *n* goes to infinity and $p_{n}$ tends to zero. A key idea for the proof of the consistency is that we obtain the convergence rate of the Newton iterative sequence for solving the estimator. The asymptotic normality is proved by applying Taylor expansions to a series of functions constructed from estimating equations and showing that remainder terms in the expansions are asymptotically neglected. Although each pair of subjects is assumed to have a comparison with the same probability $p_{n}$, our proof strategy can be easily extended to the case with different comparison probabilities at the order of $p_{n}$. Third, we use the Thurstone model to illustrate the unified theoretical results. Further extensions to a fixed sparse comparison graph in [14] are also derived. Numerical studies and real data analysis illustrate our theoretical findings.

The rest of this paper is organized as follows. In Section 2, we present the moment estimation. In Section 3, we present the consistency and asymptotic normality of the moment estimator. We illustrate our unified results with one application in Section 4. We extend the asymptotic results to a fixed comparison graph in Section 5. In Section 6, we carry out simulations and give real data analysis. We give a summary and further discussion in Section 7. The proofs of the main results are relegated to Appendix A. The proofs of supported lemmas are relegated to Appendix B.

## 2. Moment Estimation

Assume that n+1 subjects that are labeled as “0,…,n”, are compared in pairs repeatedly. Let $t_{ij}$ be the times that subject *i* compares with subject *j* and $a_{ij}$ be the times that subject *i* wins subject *j* out of $t_{ij}$ comparisons. As a result, $a_{ij}+a_{ji}=t_{ij}$. By convention, define $t_{ii}=0$ and $a_{ii}=0$. The comparison matrix ${\left(t_{ij}\right)}_{\left(n+1)\times (n+1\right)}$ is generated from an Erdös–Rényi comparison graph, where $t_{ij}$ follows a binomial distribution $Bin(T,p_{n})$ with $p_{n}$ measuring the sparsity of comparisons. More generally, $t_{ij}∼Bin\left(T_{ij},p_{n}\right)$. We set $T_{ij}$ to be the same for ease of exposition. Recall that $\beta_{0},\ldots ,\beta_{n}$ are the merit parameters of subjects 0,…,n. The probability in Model (1) implies that the winning probability only depends on the difference in merit between two subjects. For the identification of models, we normalize $\beta_{i},i=0,1,\ldots ,n$ by setting $\beta_{0}=0$ as in [10]. We assume that all paired comparisons are independent and $a_{ij}$ follows a binomial distribution $Bin(t_{ij},p_{ij})$ conditional on $t_{ij}$.

Let $a_{i}=\sum_{j=0}^{n}a_{ij}$ be the total wins of subject *i* and $a={\left(a_{1},\ldots ,a_{n}\right)}^{⊤}$. To motivate the estimating equations, we compare the maximum likelihood equation and the moment equation under the Thurstone model described in Section 4. The maximum likelihood equations are

$$\sum_{j\neq i}\left[\frac{a_{ij}\phi \left(\beta_{i}-\beta_{j}\right)}{\Phi (\beta_{i}-\beta_{j})}-\frac{\left(t_{ij}-a_{ij}\right)\phi \left(\beta_{i}-\beta_{j}\right)}{1-\Phi (\beta_{i}-\beta_{j})}\right]=0,\;\;i=1,\ldots ,n.$$

where ϕ(·) is the density function of the standard normality and Φ(·) is its distribution function. The corresponding moment equations are

$$a_{i}=\sum_{j\neq i}t_{ij}\Phi \left(\beta_{i}-\beta_{j}\right),\;\;i=1,\ldots ,n.$$

We can see that the latter is simpler and easier to compute. On the other hand, it is natural to rank subjects according to their scores. Thus, we use the moment estimation here. When F(·) in Model (1) belongs to the exponential family distributions, both are the same.

Write μ(·) as the expectation of F(·) and $\mu_{ij}\left(\beta \right)=\mu \left(\beta_{i}-\beta_{j}\right)$. Then, the estimating equations are

(2) $$a_{i}=\sum_{j\neq i}t_{ij}\mu_{ij}\left(\beta \right),\;\;i=1,\ldots ,n.$$

The solution to the above equations is the moment estimator denoted by $\hat{\beta }={\left({\hat{\beta }}_{1},\ldots ,{\hat{\beta }}_{n}\right)}^{⊤}$ and ${\hat{\beta }}_{0}=0$. Let

$$\varphi \left(\beta \right)={\left(\sum_{j\neq 1}t_{ij}\mu_{1j}\left(\beta \right),\ldots ,\sum_{j\neq n}t_{nj}\mu_{nj}\left(\beta \right)\right)}^{⊤}.$$

If $\varphi \left(\beta \right):R^{n}\to \left(0,\infty \right)$ is a one-to-one mapping, then $\hat{\beta }$ exists and is unique, i.e., $\hat{\beta }=\varphi^{-1}\left(a\right)$. When $\varphi^{-1}$ does not exist (i.e., φ is not one-to-one), any solution $\hat{\beta }$ of Equation (2) is a moment estimator of β. The Newton–Raphson algorithm can be used to solve Equation (2). Moreover, the R language provides the package “BradleyTerry2” to solve the estimator in the Bradley–Terry model.

We discuss the existence of $\hat{\beta }$ from the viewpoint of graph connection. If the comparison graph with the matrix ${\left(t_{ij}\right)}_{i,j=0,\ldots ,n}$ as its adjacency matrix is not connected, then there are two empty sets such that there are no comparisons between subjects in the first set and those in the second. In this case, there is no basis for ranking subjects in the first set and those in the second set. Further, a necessary condition for the existence of $\hat{\beta }$ is that the directed graph $G_{n}$ with the win–loss matrix $A=(a_{ij})$ as its adjacency matrix is strongly connected. In other words, for every partition of the subjects into two nonempty sets, a subject in the second set beats a subject in the first at least once. To see this, assume that there are two empty sets $B_{1}$ and $B_{2}$ such that all subjects in $B_{1}$ win all comparisons with subjects in $B_{2}$. Without loss of generality, we set $B_{1}=\left\{0,\ldots ,m\right\}$ and $B_{2}=\left\{m+1,\ldots ,n\right\}$ with 0≤m<n, where $a_{ij}=t_{ij}$ for $i\in B_{1}$ and $j\in B_{2}$. By summing $a_{i}$ over i=0,…,m, we have

$$\sum_{i=0}^{m}a_{i}=\sum_{i=0}^{m}\sum_{j=0}^{m}t_{ij}\mu \left(\beta_{i}-\beta_{j}\right)+\sum_{i=0}^{m}\sum_{j=m+1}^{n}t_{ij}\mu \left(\beta_{i}-\beta_{j}\right).$$

Because $a_{i}$ is a sum of $a_{ij}$, j=0,…,n, and $\mu \left(\beta_{i}-\beta_{j}\right)+\mu \left(\beta_{j}-\beta_{i}\right)=1$, we have

$$\sum_{i=0}^{m}\sum_{j=m+1}^{n}a_{ij}=\sum_{i=0}^{m}\sum_{j=m+1}^{n}t_{ij}\mu \left(\beta_{i}-\beta_{j}\right).$$

Because $a_{ij}=t_{ij}$ for i=0,…,m and j=m+1,…,n and at least such one $t_{ij}>0$, it must be $\mu (\beta_{i}-\beta_{j})=1$ when $t_{ij}>0$ in order to guarantee both sides in the above equation to be equal. In this case, at least one such difference $\beta_{i}-\beta_{j}$ must go to infinity such that the moment estimate does not exist. The strong connection of $G_{n}$ is also sufficient for guaranteeing the existence of the MLE in the Bradley–Terry model [19] in which the moment estimator is equal to the MLE. It is interesting to see whether the strong connection of $G_{n}$ is sufficient to guarantee the existence of $\hat{\beta }$ in a general model. In the next section, we will show that $\hat{\beta }$ exists with probability approaching one under some mild conditions.

## 3. Asymptotic Properties

In this section, we present the consistency and asymptotic normality of the moment estimator. We first introduce some notations. For a subset $C\subset R^{n}$, let $C^{0}$ and $\bar{C}$ denote the interior and closure of *C*, respectively. For a vector $x={\left(x_{1},\ldots ,x_{n}\right)}^{⊤}\in R^{n}$, denote ∥x∥ by a vector norm with the $\ell_{\infty }$-norm, ${∥x∥}_{\infty }=\max_{1\leq i\leq n}\left|x_{i}\right|$, and the $\ell_{1}$-norm, ${∥x∥}_{1}=\sum_{i}\left|x_{i}\right|$. Let $B\left(x,\epsilon \right)=\{y:∥x-y{∥}_{\infty }\leq \epsilon \}$ be an ϵ-neighborhood of *x*. For an n×n matrix $J=(J_{ij})$, let ${∥J∥}_{\infty }$ denote the matrix norm induced by the $\ell_{\infty }$-norm on vectors in $R^{n}$, i.e.,

$${∥J∥}_{\infty }=\max_{x\neq 0}\frac{{∥Jx∥}_{\infty }}{{∥x∥}_{\infty }}=\max_{1\leq i\leq n}\sum_{j=1}^{n}\left|J_{ij}\right|,$$

and let ∥J∥ be a general matrix norm. Define the matrix maximum norm: ${∥J∥}_{\max}=\max_{i,j}\left|J_{ij}\right|$. We use the superscript “*” to denote the true parameter under which the data are generated. When there is no ambiguity, we omit the superscript “*”.

Recall that μ(·) is the expectation of F(·). We assume that μ(·) is a continuous function with the third derivative. Write $\mu^{\prime }$ and $\mu^{\prime \prime }$ as the first and second derivatives of μ(π) on π, respectively. Let $\epsilon_{n}$ be a small positive number. When $\beta \in B(\beta^{*},\epsilon_{n})$, we assume that there are three positive numbers, $b_{n0},b_{n1},b_{n2}$, such that

(3a) $$\begin{array}{c} \left[\min_{i,j}\mu^{\prime }\left(\pi_{ij}\right)\right]\cdot \left[\max_{i,j}\mu^{\prime }\left(\pi_{ij}\right)\right]>0, \end{array}$$

(3b) $$\begin{array}{c} b_{n0}\leq \min_{i,j}|\mu^{\prime }\left(\pi_{ij}\right)|\leq \max_{i,j}\left|\mu^{\prime }\left(\pi_{ij}\right)\right|\leq b_{n1}, \end{array}$$

(3c) $$\begin{array}{c} \max_{i,j}\left|\mu^{\prime \prime }\left(\pi_{ij}\right)\right|\leq b_{n2}, \end{array}$$

where $\pi_{ij}:=\beta_{i}-\beta_{j}$.

We use the Bradley–Terry model to illustrate the above inequalities, where $\mu \left(x\right)=e^{x}/\left(1+e^{x}\right)$. A direct calculation gives that

$$\mu^{\prime }\left(x\right)=\frac{e^{x}}{{\left(1+e^{x}\right)}^{2}},\;\;\mu^{\prime \prime }\left(x\right)=\frac{e^{x}\left(1-e^{x}\right)}{{\left(1+e^{x}\right)}^{3}}.$$

It is easy to show that

(4) $$b_{n0}=\frac{e^{2∥\beta^{*}{∥}_{\infty }+2\epsilon_{n}}}{{\left(1+e^{2∥\beta^{*}{∥}_{\infty }+2\epsilon_{n}}\right)}^{2}}\leq |\mu^{\prime }\left(x\right)|\leq b_{n1}=\frac{1}{4},\;\;\left|\mu^{\prime \prime }\left(x\right)\right|\leq b_{n2}=\frac{1}{4}.$$

If $\epsilon_{n}=o\left(1\right)$, then $1/b_{n0}=O\left(e^{2∥\beta^{*}{∥}_{\infty }}\right)$.

### 3.1. Consistency

To establish the consistency of $\hat{\beta }$, let us first define a system of functions:

(5) $$H_{i}\left(\beta \right)=\sum_{j=0}^{n}t_{ij}\mu_{ij}\left(\beta \right)-a_{i},\;\;i=0,\ldots ,n,$$

and $H\left(\beta \right)={\left(H_{1}\left(\beta \right),\ldots ,H_{n}\left(\beta \right)\right)}^{⊤}$. It is clear that $H(\hat{\beta })=0$. Let $H^{\prime }\left(\beta \right)$ be the Jacobian matrix of H(β) on the parameter β. The asymptotic behavior of $\hat{\beta }$ depends crucially on the inverse of $H^{\prime }\left(\beta \right)$. For convenience, denote $H^{\prime }\left(\beta \right)$ as $V={\left(v_{ij}\right)}_{i,j=1,\ldots ,n}$, where

$$v_{ij}=-t_{ij}\mu^{\prime }\left(\pi_{ij}\right),\;i\neq j,\;\;v_{ii}=\sum_{j=0,j\neq i}^{n}t_{ij}\mu^{\prime }\left(\pi_{ij}\right).$$

Define

$$v_{i0}=v_{0i}:=\sum_{j=0,j\neq i}^{n}v_{ij}-v_{ii}=-t_{0i}\mu^{\prime }\left(\pi_{0i}\right),\;\;i=1,\ldots ,n,\;\;v_{00}=\sum_{j=1}^{n}t_{0j}\mu^{\prime }\left(\pi_{0j}\right).$$

When $\beta \in B(\beta^{*},\epsilon_{n})$ and $\min_{i,j}\mu^{\prime }\left(\pi_{ij}\right)>0$, in view of inequality (3b), the entries of *V* satisfy the following inequalities:

(6) $$\begin{array}{cc} \mathrm{if}\;t_{i0}>0,\;\; & t_{i0}b_{n0}\leq v_{ii}+\sum_{j=1,j\neq i}^{n}v_{ij}\leq t_{i0}b_{n1},\;\;i=1,\ldots ,n,\\ \mathrm{if}\;t_{ij}>0,\;\; & t_{ij}b_{n0}\leq -v_{ij}\leq t_{ij}b_{n1},\;\;i,j=1,\ldots ,n;i\neq j. \end{array}$$

Without loss of generality, we assume that $\min_{i\neq j}\mu^{\prime }\left(\pi_{ij}\right)>0$ when $\beta \in B(\beta^{*},\epsilon_{n})$ hereafter (otherwise, we redefine $H_{i}\left(\beta \right)=a_{i}-\sum_{j\neq i}\mu_{ij}\left(\beta \right)$ and repeat a similar process). Our strategy for the proof of consistency crucially depends on the existence of the inverse of *V*, which requires that *V* is a full rank matrix. It is easy to show that *V* is positively semi-definite. Thus, if *V* has a full rank, then *V* must be positively definite. The following lemma assures the existence of the inverse of *V*.

**Lemma** **1.** *Assume that $\min_{i,j}\mu_{ij}^{\prime }\left(\beta \right)>0$. With probability at least $1-{\left(1-p_{n}\right)}^{nT}$, $H^{\prime }\left(\beta \right)$ is positively definite.*

Because log(1−x)≤−x when x∈(0,1), we have

$$e^{nT\log(1-p_{n})}\leq e^{-p_{n}Tn}.$$

The probability of the nonexistence of $V^{-1}$ is less than $e^{-p_{n}Tn}$, going exponentially fast to zero. Generally, the inverse of *V* does not have a closed form. Ref. [20] proposed to approximate the inverse of *V*, $V^{-1}$, by the matrix $S={\left(s_{ij}\right)}_{n\times n}$, where

(7) $$s_{ij}=\frac{\delta_{ij}}{v_{ii}}+\frac{1}{v_{00}}.$$

In the above equation, $\delta_{ij}=1$ if i=j; otherwise, $\delta_{ij}=0$. By extending the proof of [20] to the sparse case, the upper bound of the approximate error $∥V^{-1}{-S∥}_{\max}$ is given in Lemma A2.

Recall that the main idea of the proof of the consistency in the Bradley–Terry model [10,14] contains two parts. Let ${\hat{u}}_{i}=e^{{\hat{\beta }}_{i}}$, $u_{i}=e^{\beta_{i}}$, $i_{0}=\arg\max_{i}{\hat{u}}_{i}/u_{i}$ and $i_{1}=\arg\min_{i}{\hat{u}}_{i}/u_{i}$. Since ${\hat{u}}_{0}/u_{0}=1$, it suffices to show that the ratio of subject $i_{0}$, ${\hat{u}}_{i_{0}}/u_{i_{0}}$, and the ratio of $i_{1}$, ${\hat{u}}_{i_{1}}/u_{i_{1}}$ are very close. With the nice mathematical properties of the logistic function $\mu \left(x\right)=e^{x}/\left(1+e^{x}\right)$, the first part is to show that there are a number of subjects satisfying the following inequalities:

(8) $$b\sum_{\left\{j:t_{i_{0},j}>0\right\}}\left({\hat{u}}_{i_{0}}/u_{i_{0}}-{\hat{u}}_{j}/u_{j}\right)\leq c,\;\;b\sum_{\left\{j:t_{i_{1},j}>0\right\}}\left({\hat{u}}_{j}/u_{j}-{\hat{u}}_{i_{0}}/u_{i_{0}}\right)\leq c,$$

where *b* and *c* are certain numbers. The second part is to eliminate common terms ${\hat{u}}_{j}/u_{j}$ based on the condition that the number of the common neighbors between any two subjects, $\min_{i,j}\#\left\{k:t_{ik}>0,t_{jk}>0\right\}$, is at least τn, where τ=1 in [10] and τ∈(0,1) in [14]. In the Erdös–Rényi comparison graph, [13] further showed that there is at least one subject with its ratio close to both ${\hat{u}}_{i_{0}}/u_{i_{0}}$ and ${\hat{u}}_{i_{1}}/u_{i_{1}}$.

The aforementioned strategies for the proof of consistency are built on the the premise of the existence of the MLE, which is guaranteed by the necessary and sufficient condition that the directed graph with the win–loss matrix as its adjacency matrix is strongly connected [19]. As discussed before, it may be difficult to find the minimal sufficient condition to guarantee the existence of $\hat{\beta }$ in general paired comparison models. To overcome this difficulty, we aim to obtain the convergence rate of the Newton iterative sequence for solving Equation (2). Under the well-known Newton–Kantorich conditions, the Newton iterative sequence converges, and its limiting point is the solution. We apply an adjusted version of the Newton–Kantorich theorem in [21] to this end, which not only guarantees the existence of the solution but also gives an optimal error bound for the Newton iterative sequence.

Now, we formally state the consistency result.

**Theorem** **1.** *Assume that Conditions* (3a)*,* (3b) *and* (3c) *hold. If $b_{n1}^{4}b_{n2}/\left(b_{n0}^{6}p_{n}^{4}\right)=o\left({\left(n/\log n\right)}^{1/2}\right)$, then $\hat{\beta }$ exists with probability approaching one and is uniformly consistent in the sense that*

(9) $$∥\hat{\beta }-\beta^{*}{∥}_{\infty }=O_{p}\left(\frac{b_{n1}^{2}}{b_{n0}^{3}p_{n}^{2}}\sqrt{\frac{\log n}{n}}\right)=o_{p}\left(1\right).$$

To see how small $p_{n}$ could be, we consider a special case that $\beta^{*}$ is a constant vector, in which $b_{n0},b_{n1}$ and $b_{n2}$ are also constants. According to the above theorem, if $p_{n}>O\left({\left(\log n/n\right)}^{1/8}\right)$, then $∥\hat{\beta }-\beta^{*}{∥}_{\infty }=O_{p}\left(p_{n}^{-2}{\left(\log n/n\right)}^{1/2}\right)$.

### 3.2. Asymptotic Normality of $\hat{\beta }$

We establish the asymptotic distribution of $\hat{\beta }$ by characterizing its asymptotical representation. In detail, we apply a second-order Taylor expansion to $H(\hat{\beta })$ and find that $\hat{\beta }-\beta^{*}$ can be represented as the sum of a main term $V^{-1}\left(a-E^{*}a\right)$ and an asymptotically neglected remainder term, where $E^{*}$ denotes the conditional expectation conditional on $\left\{t_{ij}:i,j=0,\ldots ,n\right\}$. Because $V^{-1}$ does not have a closed form, we use the matrix *S* defined in (7) to approximate it. We formally state the asymptotic normality of $\hat{\beta }$ as follows.

**Theorem** **2.** *Let $V=\partial H(\beta^{*})/\partial \beta$ and $U=\left(u_{ij}\right):=\mathrm{Var}\left(a|t_{ij},0\leq i,j\leq n\right)$. If $b_{n2}b_{n1}^{6}b_{n0}^{-9}p_{n}^{-6}=o\left(n^{1/2}/\log n\right)$, then for fixed k, the vector $\left(\left({\hat{\beta }}_{1}-\beta^{*}\right),\ldots ,\left({\hat{\beta }}_{k}-\beta_{k}^{*}\right)\right)$ follows a k-dimensional multivariate normal distribution with mean zero and the covariance matrix $\Sigma ={\left(\sigma_{ij}\right)}_{k\times k}$, where*

(10) $$\sigma_{ij}=\frac{\delta_{ij}u_{ii}}{v_{ii}^{2}}+\frac{u_{00}}{v_{00}^{2}}.$$

**Remark** **1.** *If U=V, then $\sigma_{ij}$ is equal to $\delta_{ij}/v_{ii}+1/v_{00}$. When F(·) belongs to the exponential family distribution (e.g., the Bradley–Terry model), U is identical to V. If U≠V, then the asymptotic variance of ${\hat{\beta }}_{i}$ is involved with an additional factor $u_{ii}$. The asymptotic variance of ${\hat{\beta }}_{i}$ is on the order of ${\left(np_{n}\right)}^{-1/2}$ if $\beta^{*}$ is bounded above by a constant.*

## 4. Application to the Thurston Model

In this section, we illustrate the unified theoretical result by the application to the Thurston model.

The original Thurston model has a variance $\sigma^{2}$ in the normal distribution, i.e., the probability that subject *i* is preferred over *j* is $F(\left(\beta_{i}-\beta_{j}\right)/\sigma )$. Since the merit parameters are scale invariable, we simply set σ=1 hereafter. Recall that $\phi \left(x\right)={\left(2\pi \right)}^{1/2}e^{-x^{2}/2}$ is the standard normal density function and $\Phi \left(x\right)=\int_{-\infty }^{x}\phi \left(x\right)dx$ is the distribution function of the standard normality. In the Thurston model, μ(x)=Φ(x). Then,

$$\mu^{\prime }\left(x\right)=\phi \left(x\right),\mu^{\prime \prime }\left(x\right)=\frac{x}{\sqrt{2\pi }}e^{-x^{2}/2}.$$

Since $\phi \left(x\right)={\left(2\pi \right)}^{1/2}e^{-x^{2}/2}$ is an decreasing function on |x|, we have when $\left|x\right|\leq Q_{n}$,

$$\frac{1}{2\pi }e^{-Q_{n}^{2}/2}\leq \phi \left(x\right)\leq \frac{1}{2\pi }.$$

Let $h\left(x\right)=xe^{-x^{2}/2}$. Then, $h^{\prime }\left(x\right)=\left(1-x^{2}\right)e^{-x^{2}/2}$. Therefore, when x∈(0,1), h(x) is an increasing function on its argument *x*; when x∈(1,∞), h(x) is an decreasing function on *x*. As a result, h(x) attains its maximum value at x=1 when x>0. Since h(x) is a symmetric function, we have $\left|h\left(x\right)\right|\leq e^{-1/2}\approx 0.6$. Therefore,

$$b_{n0}=\frac{1}{2\pi }e^{-(∥\beta^{*}{{∥}_{\infty }+\epsilon_{n})}^{2}/2},b_{n1}=\frac{1}{2\pi },b_{n2}={\left(2\pi e\right)}^{-1/2}.$$

In view of Theorems 1 and 2, we have the following corollary.

**Corollary** **1.** *If $b_{n1}^{4}b_{n2}/\left(b_{n0}^{6}p_{n}^{2}\right)=o\left({\left(n/\log n\right)}^{1/2}\right)$ and $p_{n}>24\log n/n$, then $\hat{\beta }$ exists with probability approaching one and is uniformly consistent in the sense that*

(11) $$∥\hat{\beta }-\beta^{*}{∥}_{\infty }=O_{p}\left(\frac{b_{n1}^{2}}{b_{n0}^{3}p_{n}}\sqrt{\frac{\log n}{n}}\right)=o_{p}\left(1\right).$$

*Let $V=\partial H(\beta^{*})/\partial \beta$. If $b_{n2}b_{n1}^{6}b_{n0}^{-9}=o(n^{1/2}/\log n)$, then for fixed k, the vector $\left(\left({\hat{\beta }}_{1}-\beta^{*}\right),\ldots ,\left({\hat{\beta }}_{k}-\beta_{k}^{*}\right)\right)$ follows a k-dimensional multivariate normal distribution with mean zero and the covariance matrix $\Sigma ={\left(\sigma_{ij}\right)}_{k\times k}$ defined at* (10)*.*

## 5. Extension to a Fixed Sparse Design

In some applications such as sports, the comparison graph may be fixed, not random. For example, in the regular season of the National Football League (NFL), games are scheduled in advance. More specially, there are 32 teams in the 2 conferences of the NFL that are divided into 8 divisions, each consisting of 4 teams. In the regular season, each team plays 16 matches, 6 within the division and 10 between the divisions. Motivated by the design, ref. [14] proposed a sparse condition to control the length from one subject to another subject with 2 or 3:

$$\tau_{n}:=\min_{0\leq i<j\leq n}\frac{\#\{k:t_{ik}>0,t_{jk}>0\}}{t}.$$

That is, $\tau_{n}$ is the minimum ratio of the total number of paths between any *i* and *j* with length 2 or 3. Under the Erdös–Rényi comparison graph, there are similar sparsities. Specifically, the set of common neighbors of any two subjects *i* and *j* has at least the following size:

$$\#\left\{k:t_{ik}>0,t_{jk}>0\right\}\geq \frac{1}{2}\left(n-1\right)p_{n}^{2},$$

with a probability of at least 1−O(1/n) if $np_{n}^{2}\geq 24\log n$; see (A12) in the proof of A2.

We assume that if two subjects have comparisons, they are compared *T* times, in accordance with the aforementioned setting for easy of exposition. Similar to Lemma A2, the approximate error of using *S* to approximate $V^{-1}$ is

$$∥V^{-1}{-S∥}_{\max}\leq \frac{2T^{2}b_{n1}^{2}\rho_{\max}}{b_{n0}^{3}\tau_{n}^{3}n^{2}},$$

where $\rho_{\max}=t_{\max}/n$ and $t_{\max}=\max_{i}t_{i}$. With similar lines of argument as in the proofs of Theorems 1 and 2, we have the following theorem, whose proof is omitted.

**Theorem** **3.** *Assume that conditions* (3a)*,* (3b) *and* (3c) *hold. If $b_{n1}^{4}b_{n2}/\left(b_{n0}^{6}\tau_{n}^{2}\right)=o\left({\left(n/\log n\right)}^{1/2}\right)$, then $\hat{\beta }$ exists with probability approaching one and is uniformly consistent in the sense that*

(12) $$∥\hat{\beta }-\beta^{*}{∥}_{\infty }=O_{p}\left(\frac{b_{n1}^{2}}{b_{n0}^{3}\tau_{n}}\sqrt{\frac{\log n}{n}}\right)=o_{p}\left(1\right).$$

*Let $V=\partial H(\beta^{*})/\partial \beta$ and $U=\left(u_{ij}\right):=\mathrm{Var}\left(a|t_{ij},0\leq i,j\leq n\right)$. If $b_{n2}b_{n1}^{6}b_{n0}^{-9}\tau_{n}^{-3}=o\left(n^{1/2}/\log n\right)$, then for fixed k, the vector $\left(\left({\hat{\beta }}_{1}-\beta^{*}\right),\ldots ,\left({\hat{\beta }}_{k}-\beta_{k}^{*}\right)\right)$ follows a k-dimensional multivariate normal distribution with mean zero and the covariance matrix $\Sigma ={\left(\sigma_{ij}\right)}_{k\times k}$, where $\sigma_{ij}$ is given in* (7)*.*

## 6. Numerical Studies

In this section, we evaluate the asymptotic results of the moment estimator in the Thurston model through simulation studies and a real data example.

### 6.1. Simulation Studies

We carry out simulations to evaluate the finite sample performance of the moment estimator in the Thurston model. We set T=1, which means that any pair has one comparison with probability $p_{n}$ and no comparison with probability $1-p_{n}$. Let *c* be a constant. We set the merit parameters to be a linear form, i.e., $\beta_{i}^{*}=ic\log n/n$ for i=1,…,n, where $\beta_{0}^{*}=0$. We considered four different values for *c* as c=0.3,0.5,0.8. By allowing $∥\beta^{*}{∥}_{\infty }$ to grow with *n*, we intended to assess the asymptotic properties under different asymptotic regimes.

In order to see how small $p_{n}$ could be, we first evaluate the fail frequency that the “win–loss” graph $G_{n}$ is strongly connected, which is the necessary condition to guarantee the moment estimator exists. We set *c* to be fixed with c=0.4. The results are shown in Table 1 with 1000. We can see that the necessary condition did not hold in each simulation when $p_{n}=\log n/n$, while it holds with almost 100% frequency when $p_{n}={\left(\log n/n\right)}^{1/2}$. This shows that it is necessary to control the rate of $p_{n}$ tending to zero.

Based on Theorem 2, ${\hat{\xi }}_{ij}=\left[{\hat{\beta }}_{i}-{\hat{\beta }}_{j}-\left(\beta_{i}^{*}-\beta_{j}^{*}\right)\right]/{\left({\hat{u}}_{ii}/{\hat{v}}_{ii}^{2}+{\hat{u}}_{00}/{\hat{v}}_{00}^{2}\right)}^{1/2}$ converges in distribution to the standard normality, where ${\hat{u}}_{ii}$ and ${\hat{v}}_{i,i}$ are the estimates of $u_{ii}$ and $v_{ii}$ by replacing $\beta^{*}$ with $\hat{\beta }$. Therefore, we assessed the asymptotic normality of ${\hat{\xi }}_{ij}$ via the coverage probability of the 95% confidence interval and the length of the confidence interval. The times that $\hat{\beta }$ failed to exist were also recorded. Two values, n=100 and n=200, were considered for the number of subjects. Each simulation was repeated 10,000 times.

The simulation results are shown in Table 2. When $p_{n}={\left(\log n/n\right)}^{1/4}$, all simulated coverage probabilities are very close to the target level 95%. On the other hand, when $p_{n}={\left(\log n/n\right)}^{1/2}$, they are a little lower than the normal level in the case n=100 and are very close to 95% in the case n=200. The length of the confidence interval decreases as *n* increases, which qualitatively agrees with the theory. It is also expected that the length of the confidence interval increases as $p_{n}$ decreases when *n* is fixed. Another phenomenon is that the length of the confidence interval under three distinct *c* has little difference when *n* and $p_{n}$ are fixed.

### 6.2. A Real Data Example

We use the 2018 NFL regular season data as an illustrative example, which is available from https://www.espn.com/nfl/schedule/_/year/2018 (accessed on 20 August 2025). The NFL league consists of thirty-two teams that are divided evenly into two conferences, and each conference has four divisions that have four teams each. In the regular season, each team plays with three intra-division teams (each twice) and ten games with ten inter-division teams (each once). As discussed in [14], the design of the NFL regular season satisfies the sparsity condition of the fixed comparison graph, where $\tau_{n}=1/16$. We removed two ties before our analysis. The fitted merits that were obtained from fitting the Thurstone model for the remaining data are given in Table 3, where we used “Arizona Cardinals” with the smallest number of wins as the baseline (with ${\hat{\beta }}_{0}=0$).

It is interesting to compare the ordering of six playoff seeds of the two conferences with the NFL rules with the ordering by their fitted merits in Table 3. The NFL rules are based on the regular season won–lost percentage record (PCT) and can be briefly summarized as follows: the teams in each division with the best PCT are seeded one through four; another two teams from each conference are seeded five and six based on their PCT. The six playoff seeds in the American Football Conference from No. 1 to No. 6 based on the PCT are Kansas City Chiefs, New England Patriots, Pittsburgh Steelers, Houston Texans, Los Angeles Chargers, and Indianapolis Colts, while the selected teams based on the fitted merits are Kansas City Chiefs, New England Patriots, Houston Texans, Baltimore Ravens, Los Angeles Chargers, and Pittsburgh Steelers. The corresponding six playoff seeds in the National Football Conference based on the PCT are Los Angeles Rams, New Orleans Saints, Chicago Bears, Washington Redskins, Seattle Seahawks, and Carolina Panthers, while the selected teams base on the fitted merits are Los Angeles Rams, New Orleans Saints, Chicago Bears, Dallas Cow boys, Seattle Seahawks, and Philadelphia Eagles. As we can see, the selected top three teams based on the PCT and the fitted merits in each conference are the same, and the selected teams from No. 4, No. 5 and No. 6 are not all the same.

## 7. Summary and Discussion

We have presented the moment estimation based on the scores of subjects in the paired comparison model under sparse comparison graphs. We have established the uniform consistency and asymptotic normality of the moment estimator. The consistency is shown by obtaining the convergence rate of the Newton iterative sequence. This leads to a condition on the sparsity parameter $p_{n}$ requiring that $p_{n}\geq O\left({\left(\log n/n\right)}^{1/8}\right)$ if $\beta^{*}$ is a constant vector. We note that this condition looks much stronger than that in the Bradley–Terry model in [13]. Since we consider a general model, it would seem to be suitable that a more severe condition is imposed. On the other hand, the condition imposed on $b_{n0}$ may not be the best possible condition. In particular, the conditions for guaranteeing the asymptotic normality seem stronger than those needed for the consistency. Note that the asymptotic behavior of the moment estimator depends not only on $b_{n0}$ but also on the configuration of all parameters. It would be of interest to investigate whether these conditions could be relaxed.

In this paper, we assume that given the comparison graph, all paired comparisons are independent. Note that the moment equation holds regardless of whether comparisons are independent.

When comparisons are not independent, the moment estimation still works. The consistency result in Theorem 1 still holds as long as there is the same order of the upper bound of $a_{i}-E^{*}a_{i}$ in Lemma A4. In fact, the independence assumption is not directly used when checking our proofs. It is only used in Lemma A4 to derive the upper bound of $a_{i}-E^{*}a_{i}$ using the Hoeffding inequality. Analogously, the independence assumption is used to derive the central limit theorem of $a_{i}-E^{*}a_{i}$. In the dependence case, there are also many Hoeffding-type exponential tail inequalities (e.g., [22,23,24]) and cental limit theorems for sums of a sequence of random variables (e.g., [25,26]) to apply.

Building on the theoretical and empirical findings of this study, we identify two promising avenues for further exploration, which not only address the current limitations but also extend the moment estimation framework to more complex and practical scenarios: 1. Moment estimation for paired comparison models with dependent outcomes. This paper assumes that all paired comparison outcomes are independent, which is a standard but restrictive assumption in many real-world settings (e.g., crowdsourcing evaluations where raters may have consistent biases, or sports leagues where team performance is serially correlated). A natural extension is to develop moment estimation methods for models with dependent outcomes, such as incorporating Markovian dependence or exchangeable correlation structures. Key challenges include deriving unbiased moment equations under dependence and establishing asymptotic properties (consistency, asymptotic normality) using tools from dependent random variable theory (e.g., Hoeffding-type inequalities for associated variables [22,23,24]). 2. High-dimensional sparse paired comparison models with structured merit parameters. This study focuses on unstructured merit parameters (i.e., $\beta_{i}$ are independent). However, in many applications, merit parameters often exhibit inherent structures, such as group-level homogeneity (e.g., teams in the same sports division share similar strengths) or sparsity (e.g., only a few subjects have distinct merits in large-scale crowdsourcing). Extending the moment estimation framework to incorporate such structures (e.g., group lasso-penalized moment estimation, sparse merit parameter inference) would improve estimation efficiency. Key research questions include designing computationally feasible penalized moment equations and establishing oracle properties for the structured estimators. These directions align with the core theme of sparse paired comparison inference and address practical limitations of the current work. We believe pursuing these avenues will not only extend the theoretical scope of moment estimation but also broaden its applicability to more complex real-world problems.

## Figures and Tables

**Table 1:** The fail frequency (×100%).

$p_{n}$	n=100	n=500	n=1000
${\left(\log n/n\right)}^{1/2}$	0.5	0	0
${\left(\log n/n\right)}^{2/3}$	25.8	0.2	0
logn/n	100	100	100

**Table 2:** The reported values are the coverage frequency (×100%) for $\beta_{i}-\beta_{j}$ for a pair (i,j)/length of the confidence interval/fail probabilities (×100%).

$p_{n}={\left(\log n/n\right)}^{1/4}$				
n	i	c=0.2	c=0.5	c=0.8
100	(1,2)	94.5/1.04/0	94.62/1.06/0	94.62/1.06/0
	(49,50)	94.97/1.04/0	94.34/1.04/0	94.34/1.04/0
	(99,100)	95.12/1.04/0	94.99/1.06/0	94.99/1.06/0
200	(1,2)	95.18/0.78/0	94.71/0.79/0	94.71/0.79/0
	(99,100)	95.14/0.78/0	94.49/0.78/0	94.49/0.78/0
	(199,200)	94.82/0.78/0	94.64/0.79/0	94.64/0.79/0
$p_{n}={\left(\log n/n\right)}^{1/2}$				
**n**	i	c=0.2	c=0.4	c=0.6
100	(1,2)	93.28/1.58/0.19	93.48/1.60/0.49	93.48/1.60/1.15
	(49,50)	93.85/1.58/0.19	93.94/1.58/0.49	93.94/1.58/1.15
	(99,100)	93.80/1.58/0.19	93.96/1.61/0.49	93.96/1.61/1.15
200	(1,2)	94.04/1.26/0	93.89/1.28/0	93.89/1.28/0
	(99,100)	94.14/1.26/0	94.22/1.26/0	94.22/1.26/0
	(199,200)	94.38/1.26/0	93.98/1.28/0	93.98/1.28/0

**Table 3:** The fitted merit ${\hat{\beta }}_{i}$, the number of wins $a_{i}$, and the standard error ${\hat{\sigma }}_{i}$.

American Football Conference					National Football Conference				
Division	Team	${\hat{\beta }}_{i}$	$a_{i}$	${\hat{\sigma }}_{i}$	Division	Team	${\hat{\beta }}_{i}$	$a_{i}$	${\hat{\sigma }}_{i}$
East	New England Patriots	1.452	11	0.519	East	Dallas Cow boys	1.284	10	0.512
	New York Jets	0.338	4	0.530		Philadelphia Eagles	1.193	9	0.511
	Miami Dophins	0.718	7	0.509		New York Giants	0.423	5	0.514
	Buffalo Bills	0.673	6	0.514		Washington Redskins	0.756	7	0.511
North	Baltimore Ravens	1.382	10	0.514	North	Chicago Bears	1.494	12	0.532
	Cincinnati Bengals	0.769	6	0.514		Green Bay Packers	0.595	6	0.522
	Pittsburgh Steelers	1.337	9	0.52		Minnesota Vikings	1.059	8	0.523
	Cleveland Browns	0.968	7	0.519		Detroit Lions	0.579	6	0.516
South	Indianapolis Colts	1.244	10	0.512	South	New Orleans Saints	1.908	13	0.544
	Houston Texans	1.430	11	0.516		Atlanta Falcons	0.767	7	0.512
	Tennessee Titans	1.205	9	0.51		Carolina Panthers	0.840	7	0.511
	Jacksonville Jaguars	0.591	5	0.517		Tampa Bay Buccaneers	0.506	5	0.52
West	Kansas City Chiefs	1.762	12	0.537	West	Los Angeles Rams	1.963	13	0.559
	Denver Broncos	0.713	6	0.523		San Francisco 49ers	0.166	4	0.54
	Oakland Raiders	0.365	4	0.537		Seattle Seahawks	1.305	10	0.525
	Los Angeles Chargers	1.748	12	0.536		Arizona Cardinals	0	3	0.555

## Data Availability

We use the 2018 NFL regular season data as an example, which is available from https://www.espn.com/nfl/schedule/_/year/2018 (accessed on 20 August 2025).

## Appendix A

In this section, we present the proofs of the theorems.

### Appendix A.1. Preliminaries

We state two preliminary results firstly, which will be used in the proofs. The first result is the optimal error bound in the Newton method in [21] under the Kantorovich conditions [27].

**Lemma** **A1** ([21])**.** *Let X and Y be Banach spaces, D be an open convex subset of X and F: D⊆X→Y be Fréchet differentiable. Assume that, at some $x_{0}\in D$, $F^{\prime }\left(x_{0}\right)$ is invertible and that*

(A1) $$\begin{array}{c} ∥F^{\prime }{\left(x_{0}\right)}^{-1}\left(F^{\prime }\left(x\right)-F^{\prime }\left(y\right)\right)∥\leq K∥x-y∥,\;\;x,y\in D, \end{array}$$

(A2) $$\begin{array}{c} ∥F^{\prime }{\left(x_{0}\right)}^{-1}F\left(x_{0}\right)∥\leq \eta ,h=K\eta \leq 1/2,\\ \bar{S}\left(x_{0},t^{*}\right)\subseteq D,t^{*}=2\eta /\left(1+\sqrt{1-2h}\right). \end{array}$$

*Then: (1) The Newton iterates $x_{n+1}=x_{n}-F^{\prime }{\left(x_{n}\right)}^{-1}F\left(x_{n}\right)$, n≥0 are well-defined, lie in $\bar{S}\left(x_{0},t^{*}\right)$ and converge to a solution $x^{*}$ of F(x)=0.*
*(2) The solution $x^{*}$ is unique in $S(x_{0},t^{**})\cap D$, $t^{**}=\left(1+\sqrt{1-2h}\right)/K$ if 2h<1 and in $\bar{S}\left(x_{0},t^{**}\right)$ if 2h=1.*
*(3) $∥x^{*}-x_{n}∥\leq t^{*}$ if n=0 and $∥x^{*}-x_{n}∥\leq 2^{1-n}{\left(2h\right)}^{2^{n}-1}\eta$ if n≥1.*

The second result is the approximate error of using *S* to approximate $V^{-1}$, whose proof is in the Appendix B.

**Lemma** **A2.**
*If $p_{n}\geq 24\log n/n$, then for sufficiently large n with a probability of at least $1-O(n^{-1})$, we have*

$$∥V^{-1}{-S∥}_{\max}\leq \frac{12Tb_{n1}^{2}}{b_{n0}^{3}n\left(n-1\right)p_{n}^{3}}.$$

### Appendix A.2. Proof of Theorem 1

We aim to show Theorem 1 by obtaining the convergence rate of the Newton iterative sequence in view of Lemma A1, which requires us to verify the Kantovorich conditions (A1) and (A2). Condition (A1) depends on the Lipschitz continuous form of $H_{i}^{\prime }\left(\beta \right)$. Recall that $t_{\max}=\max_{i=0,\ldots ,n}t_{i}$ and $t_{\min}=\min_{i=0,\ldots ,n}t_{i}$.

**Lemma** **A3.** *Let $D=B\left(\beta^{*},\epsilon_{n}\right)\left(\subset R^{n}\right)$ be an open convex set containing the true point $\beta^{*}$. For any given set $\left\{t_{ij},0\leq i,j\leq n\right\}$, if Inequality* (3c) *holds, then*

$$\max_{i=0,\ldots ,n}∥H_{i}^{\prime }\left(x\right)-H_{i}^{\prime }{\left(y\right)∥}_{1}\leq 4b_{n2}t_{\max}{∥x-y∥}_{\infty }.$$

Moreover, Condition (A1) also depends on the magnitudes of $|a_{i}-E\left(a_{i}|t_{ij},j=0,\ldots n\right)|$, i=0,…,n, which are stated below.

**Lemma** **A4.**
*With a probability of at least 1−O(1/n), we have*

$$\max_{i=0,\ldots ,n}\left|a_{i}-E\left(a_{i}|t_{ij},j=0,\ldots ,n\right)\right|\leq \sqrt{2\log nt_{\max}}.$$

The following results are the lower bound of $t_{\min}$ and the upper bounds of $t_{\max}$ and of $\sum_{i}t_{i}$.

**Lemma** **A5.**
*(1) With probability at least $1-\left(n+1\right)\exp\left(-\frac{1}{8}nTp_{n}\right)$,*

$$t_{\min}=\min_{i=0,\ldots ,n}t_{i}\geq \frac{1}{2}nTp_{n}.$$

*(2) With probability at least $1-\left(n+1\right)\exp\left(-\frac{1}{10}nTp_{n}\right)$,*

$$t_{\max}=\max_{i=0,\ldots ,n}t_{i}\leq \frac{3}{2}nTp_{n}.$$

*(3) With probability at least $1-\exp(-\frac{1}{10}n\left(n+1\right)Tp_{n}$,*

$$\sum_{i=0}^{n}t_{i}\leq 3n\left(n+1\right)Tp_{n}.$$

We are now ready to prove Theorem 1.

**Proof of Theorem** **1.** Note that $\hat{\beta }$ is the solution to the equation H(β) = 0. We prove the consistency by obtaining the convergence rate of the Newton iterative sequence: $\beta^{\left(k+1\right)}=\beta^{\left(k\right)}-{\left[H^{\prime }\left(\beta^{\left(k\right)}\right)\right]}^{-1}H\left(\beta^{\left(k\right)}\right)$, where we set $\beta^{\left(0\right)}:=\beta^{*}$. To apply Lemma A1, we choose the convex set $D=B(\beta^{*},\epsilon_{n})$. The following calculations are based on the event $E_{n}$:

$$\begin{array}{c} \{t_{ij},0\leq i,j\leq n:\max_{i}|a_{i}-E\left(a_{i}|t_{ij},j=0,\ldots ,n\right)|\leq \sqrt{2t_{\max}\log n},\\ H^{\prime }\left(\beta \right)>0,\;\;t_{\min}\geq \frac{T}{2}np_{n},\;\;t_{\max}\leq \frac{3}{2}nTp_{n}\}. \end{array}$$

Note that $b_{n0}\leq \left|\mu_{ij}^{\prime }\left(\beta \right)\right|\leq b_{n1}$ when $\beta \in B(\beta^{*},\epsilon_{n})$. Let $V={\left(v_{ij}\right)}_{n\times n}=H^{\prime }\left(\beta^{*}\right)$. We use *S* defined in (7) to approximate $V^{-1}$ and let $W=V^{-1}-S$. We verify the Kantovorich conditions in Lemma A1 as follows. Since $\sum_{i=0}^{n}H_{i}\left(\beta \right)=0$, we have

$$\sum_{i=1}^{n}H_{i}\left(\beta \right)=-H_{0}\left(\beta \right).$$

Based on Lemma A3, we have

$$\begin{array}{ccc} & & ∥{\left[H^{\prime }\left(\beta^{*}\right)\right]}^{-1}\left[H^{\prime }\left(x\right)-H^{\prime }\left(y\right)\right]{∥}_{\infty }\\ & \leq & ∥S\left[H^{\prime }\left(x\right)-H^{\prime }\left(y\right)\right]{∥}_{\infty }+{∥W\left[H^{\prime }\left(x\right)-H^{\prime }\left(y\right)\right]∥}_{\infty }\\ & \leq & \max_{i=1,\ldots ,n}\frac{1}{v_{ii}}∥H_{i}^{\prime }\left(x\right)-H_{i}^{\prime }{\left(y\right)∥}_{1}+\frac{1}{v_{00}}∥H_{0}^{\prime }\left(x\right)-H_{0}^{\prime }{\left(y\right)∥}_{1}+{∥W∥}_{\infty }{∥H^{\prime }\left(x\right)-H^{\prime }\left(y\right)∥}_{\infty }\\ & \leq & \left[\frac{2}{b_{n0}t_{\min}}+n\cdot \frac{12b_{n1}^{2}T}{n\left(n-1\right)b_{n0}^{3}p_{n}^{3}}\right]\times 4b_{n2}t_{\max}\times {∥x-y∥}_{\infty }\\ & = & O\left(\frac{b_{n1}^{2}b_{n2}}{b_{n0}^{3}p_{n}^{2}}\right)\times {∥x-y∥}_{\infty }. \end{array}$$

Thus, we can set $K=O(b_{n1}^{2}b_{n2}b_{n0}^{-3}\eta_{n}^{-1})$ in (A1). Again, based on the event $E_{n}$, we have

$$\begin{array}{ccc} \eta & = & ∥{\left[H^{\prime }\left(\beta^{*}\right)\right]}^{-1}H\left(\beta^{*}\right){∥}_{\infty }\\ & \leq & n∥V^{-1}{-S∥}_{\max}{∥H\left(\beta^{*}\right)∥}_{\infty }+\max_{i=1,\ldots ,n}\frac{|H_{i}\left(\beta^{*}\right)|}{v_{ii}}+\frac{|H_{0}\left(\beta^{*}\right)|}{v_{00}}\\ & \leq & \left[O\left(\frac{b_{n1}^{2}}{nb_{n0}^{3}p_{n}^{3}}\right)+O\left(\frac{1}{b_{n0}t_{\min}}\right)\right]\times O\left({\left(t_{\max}\log n\right)}^{1/2}\right)\\ & = & O\left(\frac{b_{n1}^{2}}{b_{n0}^{3}p_{n}^{2}}\sqrt{\frac{\log n}{n}}\right). \end{array}$$

If

(A3) $$K\eta =O\left(\frac{b_{n1}^{4}b_{n2}}{b_{n0}^{6}p_{n}^{4}}\sqrt{\frac{\log n}{n}}\right)=o\left(1\right),$$

then this verifies Condition (A2). Based on Lemma A1, $\lim_{k}\beta^{\left(k\right)}$ exists, denoted by $\hat{\beta }$, and it satisfies

$$∥\hat{\beta }-\beta^{*}{∥}_{\infty }=O\left(\frac{b_{n1}^{2}}{b_{n0}^{3}p_{n}^{2}}\sqrt{\frac{\log n}{n}}\right).$$

Based on Lemmas 1, A2, A4 and A5, the event $E_{n}$ holds with a probability of at least $1-O(n^{-1})$ if $p_{n}\geq 24\log n/n$. Note that (A3) implies $p_{n}\geq 24\log n/n$. This completes the proof. □

### Appendix A.3. Proofs for Theorem 2

Write ${\mathrm{Var}}^{*}$ and $E^{*}$ as the conditional variance and conditional expectation given $t_{ij}$ for 0≤i,j≤n. Let $U=\left(u_{ij}\right):={\mathrm{Var}}^{*}\left(a\right)$. In the Bradley–Terry model, $U=H^{\prime }\left(\beta^{*}\right)$. Note that $a_{i}$ is a sum of $t_{i}$ independent Bernoulli random variables. Based on Lemma A5, we know that $\min_{i}t_{i}=O_{p}\left(np_{n}\right)$. Let $\sigma_{\min}=\min_{i\neq j}p_{ij}\left(1-p_{ij}\right)$. If $np_{n}\sigma_{\min}\to \infty$, then $\min_{i}u_{ii}\to \infty$. Based on the central limit theorem in the bound case, as in [28] (p. 289), if $np_{n}\sigma_{\min}\to \infty$, then $u_{ii}^{-1/2}\left\{a_{i}-E^{*}\left(a_{i}\right)\right\}$ converges in distribution to the standard normal distribution. When considering the asymptotic behaviors of the vector $\left(a_{1},\ldots ,a_{r}\right)$ with a fixed *r*, one could replace the degrees $a_{1},\ldots ,a_{r}$ by the independent random variables ${\tilde{a}}_{i}=a_{i,r+1}+\ldots +a_{in}$, i=1,…,r. Therefore, we have the following proposition.

**Proposition** **A1.**
*If $np_{n}\sigma_{\min}\to \infty$, then as n→∞, for any fixed r≥1, the components of $\left(a_{1}-E^{*}\left(a_{1}\right),\ldots ,a_{r}-E^{*}\left(a_{r}\right)\right)$ are asymptotically independent and normally distributed with variances $u_{11},\ldots ,u_{rr}$, respectively. Moreover, the first r rows of $S(a-E^{*}\left(a\right))$ are asymptotically normal with covariance matrix $\Sigma =(\sigma_{ij})$, where*

$$\sigma_{ij}=\frac{\delta_{ij}u_{ii}}{v_{ii}^{2}}+\frac{u_{00}}{v_{00}^{2}}.$$

**Lemma** **A6.**
*Let $V=H^{\prime }\left(\beta^{*}\right)$ and $W=V^{-1}-S$ and ${\mathrm{Cov}}^{*}\left(\cdot \right)=\mathrm{Cov}\left(\cdot |t_{ij},0\leq i,j\leq n\right)$. Then,*

$${\mathrm{Cov}}^{*}\left(WH^{\prime }\left(\beta^{*}\right)\right)=O_{p}\left(\frac{b_{n1}^{5}}{n^{2}b_{n0}^{6}p_{n}^{5}}\right).$$

*Further, if $U=H^{\prime }\left(\beta^{*}\right)$, then*

$${\mathrm{Cov}}^{*}\left(WH^{\prime }\left(\beta^{*}\right)\right)=O_{p}\left(\frac{b_{n1}^{2}}{n^{2}b_{n0}^{3}p_{n}^{5}}\right).$$

Now, we are ready to prove Theorem 2.

**Proof of Theorem** **2.** Let ${\hat{\pi }}_{ij}={\hat{\beta }}_{i}-{\hat{\beta }}_{j}$ and $\pi_{ij}^{*}=\beta_{i}^{*}-\beta_{j}^{*}$. Based on Theorem 1, $\hat{\beta }\in B\left(\beta^{*},\epsilon_{n}\right)$. To simplify notations, write $\mu_{ij}^{\prime }=\mu^{\prime }\left(\pi_{ij}^{*}\right)$. Based on a second-order Taylor expansion, we have

(A4) $$t_{ij}\mu \left({\hat{\pi }}_{ij}\right)-t_{ij}\mu \left(\pi_{ij}^{*}\right)=t_{ij}\mu_{ij}^{\prime }\left({\hat{\beta }}_{i}-\beta_{i}^{*}\right)-t_{ij}\mu_{ij}^{\prime }\left({\hat{\beta }}_{j}-\beta_{j}^{*}\right)+g_{ij},\;\;i\neq j,$$

where $g_{ij}$ is the second-order remainder term:

$$\begin{array}{c} g_{ij}=t_{ij}\mu^{\prime \prime }\left({\tilde{\pi }}_{ij}\right)\left[{\left({\hat{\beta }}_{i}-\beta_{i}\right)}^{2}+{\left({\hat{\beta }}_{j}-\beta_{j}\right)}^{2}-2\left({\hat{\beta }}_{i}-\beta_{i}\right)\left({\hat{\beta }}_{j}-\beta_{j}\right)\right]. \end{array}$$

In the above equation, ${\tilde{\pi }}_{ij}$ lies between $\pi_{ij}^{*}$ and ${\hat{\pi }}_{ij}$. If $b_{n1}^{4}b_{n2}b_{n0}^{-6}p_{n}^{-4}=o\left({\left(n/\log n\right)}^{1/2}\right)$, based on Theorem 1, we have

$$∥\hat{\beta }-\beta^{*}{∥}_{\infty }=O_{p}\left(\frac{b_{n1}^{2}}{b_{n0}^{3}p_{n}^{2}}\sqrt{\frac{\log n}{n}}\right).$$

Therefore, in view of (3c), $|\mu_{ij}^{\prime \prime }\left({\tilde{\pi }}_{ij}\right)|\leq t_{ij}b_{n2}$ such that

(A5) $$|g_{ij}|\leq 4b_{n2}t_{ij}{∥\hat{\beta }-\beta^{*}∥}_{\infty }^{2}.$$

Let $g_{i}=\sum_{j\neq i}g_{ij}$, i=0,…,n, and $g={\left(g_{1},\ldots ,g_{n}\right)}^{⊤}$. Then, based on Lemma A5 (2), we have

(A6) $$\max_{i=0,\ldots ,n}\left|g_{i}\right|=4b_{n2}t_{\max}\cdot O_{p}\left(\frac{b_{n1}^{4}\log n}{nb_{n0}^{6}p_{n}^{4}}\right)=O_{p}\left(\frac{b_{n1}^{4}b_{n2}\log n}{b_{n0}^{6}p_{n}^{3}}\right).$$

By writing the equation in (A4) into a matrix form, we have

(A7) $$E^{*}a-a=V\left(\hat{\beta }-\beta^{*}\right)+g.$$

Equivalently,

(A8) $$\hat{\beta }-\beta^{*}=V^{-1}\left(E^{*}a-a\right)+V^{-1}g.$$

Similarly, we have

$$\begin{array}{ccc} E^{*}a_{0}-a_{0} & = & \frac{\partial H_{0}\left(\beta^{*}\right)}{\partial \beta }\left(\hat{\beta }-\beta^{*}\right)+\frac{1}{2}{\left(\hat{\beta }-\beta^{*}\right)}^{⊤}\frac{\partial^{2}H_{0}\left(\tilde{\beta }\right)}{\partial \beta \partial \beta^{⊤}}\left(\hat{\beta }-\beta^{*}\right)\\ & = & -\sum_{i=1}^{n}v_{i0}\left({\hat{\beta }}_{i}-\beta_{i}^{*}\right)+\frac{1}{2}v_{i0}{\left({\hat{\beta }}_{i}-\beta_{i}^{*}\right)}^{2}. \end{array}$$

Therefore, based on Lemma A5 (2), we have

$$|E^{*}a_{0}-a_{0}+\sum_{i=1}^{n}v_{i0}\left({\hat{\beta }}_{i}-\beta_{i}^{*}\right)|=O_{p}\left(\frac{b_{n1}^{4}t_{\max}\log n}{nb_{n0}^{6}p_{n}^{4}}\right)=O_{p}\left(\frac{b_{n1}^{4}\log n}{b_{n0}^{6}p_{n}^{3}}\right).$$

Note that $\sum_{i=0}^{n}a_{i}-E^{*}\left(a_{i}\right)=0$. Multiplying both sides of (A7) by a row vector with all element 1 yields

$$\sum_{i=1}^{n}g_{i}=a_{0}-E^{*}a_{0}+\sum_{i=1}^{n}v_{i0}\left({\hat{\beta }}_{i}-\beta_{i}^{*}\right).$$

Therefore, we have

(A9) $$|\sum_{i=1}^{n}g_{i}|=O_{p}\left(\frac{b_{n1}^{4}\log n}{b_{n0}^{6}p_{n}^{3}}\right).$$

Based on (A6) and Lemma A2, we have

$$\begin{array}{ccc} ∥V^{-1}{g∥}_{\infty } & \leq & {∥Sg∥}_{\infty }+{∥\left(V^{-1}-S\right)g∥}_{\infty }\\ & \leq & \max_{i=1,\ldots ,n}\frac{1}{v_{ii}}|g_{i}|+\frac{1}{v_{00}}|\sum_{i=1}^{n}g_{i}|+n∥V^{-1}{-S∥}_{\max}{∥g∥}_{\infty }\\ & = & O_{p}\left(\frac{b_{n2}}{t_{\min}b_{n0}}+\frac{b_{n1}^{2}}{nb_{n0}^{3}p_{n}^{3}}\right)\times O_{p}\left(\frac{b_{n1}^{4}b_{n2}\log n}{b_{n0}^{6}p_{n}^{3}}\right)\\ & = & O_{p}\left(\frac{b_{n2}b_{n1}^{6}\log n}{nb_{n0}^{9}p_{n}^{6}}\right). \end{array}$$

If $b_{n2}b_{n1}^{6}b_{n0}^{-9}p_{n}^{-6}=o\left(n^{1/2}/\log n\right)$, then we have

(A10) $${\hat{\beta }}_{i}-\beta_{i}^{*}=V^{-1}\left(E^{*}a-a\right)+o_{p}\left(n^{-1/2}\right).$$

Consequently, in view of Lemma A6, we have

$${\hat{\beta }}_{i}-\beta_{i}^{*}={\left[S\left(E^{*}a-a\right)\right]}_{i}+o_{p}\left(n^{-1/2}\right).$$

Therefore, Theorem 2 immediately comes from Proposition A1. □

## Appendix B

In this section, we present the proofs of supported lemmas.

### Appendix B.1. Proof of Lemma 1

**Proof of Lemma** **1.** For an arbitrarily given nonzero vector $x={\left(x_{1},\ldots ,x_{n}\right)}^{⊤}\in R^{n}$, direct calculations give

$$\begin{array}{ccc} x^{⊤}Vx & = & =\sum_{i=1}^{n}x_{i}^{2}v_{ii}+\sum_{i=1}^{n}\sum_{j=1,j\neq i}^{n}x_{i}v_{ij}x_{j}\\ & = & -\sum_{i=1}^{n}\sum_{j=1,j\neq i}^{n}x_{i}^{2}v_{ij}-\sum_{i=1}^{n}x_{i}^{2}v_{i0}+\sum_{i=1}^{n}\sum_{j=1,j\neq i}^{n}x_{i}v_{ij}x_{j}\\ & = & -\frac{1}{2}\sum_{i=1}^{n}\sum_{j=1,j\neq i}^{n}{\left(x_{i}-x_{j}\right)}^{2}v_{ij}-\sum_{i=1}^{n}x_{i}^{2}v_{i0}, \end{array}$$

where the second equality is due to that $v_{ii}=-\sum_{j\neq i}v_{ij}$. Therefore, $x^{⊤}Vx=0$ if and only if

$$x_{i}v_{i0}=0,\;\;i=1,\ldots ,n,v_{ij}\left(x_{i}-x_{j}\right)=0,1\leq i\neq j\leq n.$$

Because $\mu_{ij}\left(\beta \right)\neq 0$ and $v_{ij}=t_{ij}\mu_{ij}\left(\beta \right)$ for i≠j, the above equations are identical to

$$x_{i}t_{i0}=0,\;\;i=1,\ldots ,n,t_{ij}\left(x_{i}-x_{j}\right)=0,1\leq i\neq j\leq n.$$

Let *E* be the event

$$\{{\left\{t_{ij}\right\}}_{i,j=0,i\neq j}^{n}:x_{i}t_{i0}=0,\;\;i=1,\ldots ,n,t_{ij}\left(x_{i}-x_{j}\right)=0,1\leq i\neq j\leq n\}.$$

To show Lemma 1, it is sufficient to obtain the lower bound of the probability of the event *E*. We will evaluate the probability of the event *E* under two cases: there exists some zero element in *x* and there are no zero elements in *x*. Case I: We consider there are zero elements in *x*. Let $\left\{0,x_{i_{1}},\ldots ,x_{i_{k}}\right\}$ be k+1 different distinct values in $\left\{x_{1},\ldots ,x_{n}\right\}$, $\Omega_{0}=\left\{i:x_{i}=0\right\}$ and $\Omega_{j}=\left\{q:x_{q}=x_{i_{j}}\right\}$, j=1,…,k. Since x≠0, k≥1. It is clear that

(A11) $$|\Omega_{0}\left|>0,\;\;\right|\Omega_{j}|>0,j=1,\ldots ,k,\;\;\sum_{i=0}^{k}\left|\Omega_{i}\right|=n+1,$$

where $|\Omega_{i}|$ denotes the cardinality of $\Omega_{i}$. Therefore, we have

$$\begin{array}{ccc} P(E) & = & {\left(1-p_{n}\right)}^{\sum_{j=1}^{k}\left|\Omega_{j}\right|}\times \prod_{0\leq i<j\leq k}{\left(1-p_{n}\right)}^{T|\Omega_{i}\left|\right|\Omega_{j}|}\\ & = & {\left(1-p_{n}\right)}^{\sum_{j=1}^{k}|\Omega_{j}|+\sum_{0\leq i<j\leq k}|\Omega_{i}\left|\right|\Omega_{j}|}. \end{array}$$

To obtain the lower bound of P(E), it is sufficient to solve the minimizer of $\sum_{j=1}^{k}|\Omega_{j}|+\sum_{0\leq i<j\leq k}|\Omega_{i}\left|\right|\Omega_{j}|$ under the restricted condition (A11). Let $y_{i}=\left|\Omega_{i}\right|$. Then,

$$\begin{array}{cc} \; & \sum_{j=1}^{k}y_{i}+\sum_{0\leq i<j\leq k}y_{i}y_{j}\\ = & \sum_{j=1}^{k}y_{i}+\frac{1}{2}\sum_{i=0}^{n}\sum_{j=0,j\neq i}^{k}y_{i}y_{j}\\ = & \frac{1}{2}\sum_{i=0}^{n}\sum_{j=0}^{n}y_{i}y_{j}-\frac{1}{2}\sum_{i=0}^{k}y_{i}^{2}+\sum_{j=1}^{k}y_{i}\\ = & \frac{1}{2}{\left(\sum_{i=0}^{n}y_{i}\right)}^{2}-\frac{1}{2}\sum_{i=1}^{k}{\left(y_{i}-1\right)}^{2}-\frac{1}{2}y_{0}^{2}+\frac{1}{2}k\\ = & \frac{1}{2}\left({\left(n+1\right)}^{2}+k\right)-\frac{1}{2}\left(\sum_{i=0}^{k}z_{i}^{2}\right), \end{array}$$

where $z_{0}=y_{0}$ and $z_{i}=y_{i}-1$, i=1,…,k. Under the restriction $\sum_{i}z_{i}=n+1-k>0$ and $z_{i}\geq 0$, the function $\sum_{i=0}^{k}z_{i}^{2}$ obtains its maximizer at such points z=(0,…,n+1−k,0,…,0). Therefore, we have

$$\begin{array}{cc} \; & \sum_{j=1}^{k}y_{i}+\sum_{0\leq i<j\leq k}y_{i}y_{j}\\ \geq & \frac{1}{2}\left({\left(n+1\right)}^{2}+k-{\left(n+1-k\right)}^{2}\right)=\frac{1}{2}\left(2\left(n+1\right)k+k-k^{2}\right)\\ = & \frac{1}{2}\left[-{\left(k-\frac{2(n+1)+1}{2}\right)}^{2}+{\left(n+1+\frac{1}{2}\right)}^{2}\right]. \end{array}$$

Because 1≤k≤n, the above function obtains its minimizer at k=1. That is,

$$\begin{array}{cc} \; & -{\left(k-\frac{2(n+1)+1}{2}\right)}^{2}+{\left(\left(n+1\right)+\frac{1}{2}\right)}^{2}\\ \geq & -{\left(1-\left(n+1\right)-1/2\right)}^{2}+{\left(n+1\right)}^{2}+\left(n+1\right)+1/4\\ = & 2(n+1). \end{array}$$

This shows

$$P\left(E\right)\leq {\left(1-p_{n}\right)}^{2T(n+1)}.$$

Case II: there are no zero elements in *x*. With the same notation $\Omega_{j}$ as in Case I, we have that $|\omega_{0}|=0$ and

$$\begin{array}{ccc} P(E) & = & {\left(1-p_{n}\right)}^{\sum_{j=1}^{k}\left|\Omega_{j}\right|}\times \prod_{1\leq i<j\leq k}{\left(1-p_{n}\right)}^{T|\Omega_{i}\left|\right|\Omega_{j}|}\\ & = & {\left(1-p_{n}\right)}^{T(\sum_{j=1}^{k}|\Omega_{j}|+\sum_{1\leq i<j\leq k}|\Omega_{i}||\Omega_{j}|)}. \end{array}$$

It is sufficient to obtain the minimizer of $\sum_{j=1}^{k}|\Omega_{j}|+\sum_{1\leq i<j\leq k}|\Omega_{i}\left|\right|\Omega_{j}|$. under the restriction $\sum_{i}\left|\Omega_{i}\right|=n$ and k≥1. Let $y_{i}=\left|\Omega_{i}\right|$. Then,

$$\begin{array}{ccc} \sum_{j=1}^{k}y_{i}+\sum_{1\leq i<j\leq k}y_{i}y_{j} & = & \sum_{j=1}^{k}y_{i}+\frac{1}{2}\sum_{i=1}^{n}\sum_{j=1,j\neq i}^{k}y_{i}y_{j}\\ & = & \frac{1}{2}\sum_{i=1}^{k}\sum_{j=1}^{k}y_{i}y_{j}-\frac{1}{2}\sum_{i=1}^{k}y_{i}^{2}+\left(n+1\right)\\ & = & \frac{1}{2}{\left(\sum_{i=1}^{k}y_{i}\right)}^{2}-\frac{1}{2}\sum_{i=1}^{k}y_{i}^{2}+\left(n+1\right)\\ & = & \frac{1}{2}{\left(n+1\right)}^{2}+\left(n+1\right)-\frac{1}{2}\left(\sum_{i=1}^{k}y_{i}^{2}\right). \end{array}$$

Under the restriction $\sum_{i}y_{i}=n+1>0$ and $z_{i}\geq 0$, the functions $\sum_{i=1}^{k}z_{i}^{2}$ obtain their maximizer at points z=(0,…,n+1,0,…,0). Therefore, we have

$$\sum_{j=1}^{k}y_{i}+\sum_{1\leq i<j\leq k}y_{i}y_{j}\geq \frac{1}{2}\left({\left(n+1\right)}^{2}+\left(n+1\right)-{\left(n+1\right)}^{2}\right)\geq n+1.$$

This shows

$$P\left(E\right)\leq {\left(1-p_{n}\right)}^{(n+1)T}.$$

By combining the lower bounds of P(E) under Cases I and II, we have

$$P\left(E^{c}\right)\geq 1-{\left(1-p_{n}\right)}^{(n+1)T},$$

where $E^{c}$ denotes that *V* is positively definite. □

### Appendix B.2. Proof of Lemma A2

**Proof of Lemma** **A2.** Based on Lemma 1, *V* is a positively definite matrix with a probability of at least $1-e^{-p_{n}T\left(n+1\right)}$. In what follows, we assume that *V* is positively definite such that its inverse exists. The proof proceeds two parts. The first part is to evaluate the cardinality of the set of the common neighbors of any two subjects *i* and *j*. That is, we establish the lower bound:

$$\min_{i,j}\#\left\{k:t_{ik}>0,t_{kj}>0\right\}.$$

The second part is to show such inequality [c.f. (A16)]

$$a\geq b[\sum_{\left\{k:t_{\alpha k}>0,t_{k\beta }>0\right\}}\left(z_{i\alpha }-z_{i\beta }\right)].$$

We use the method of the proof in [20] with minor modifications that simplify their proofs to show the second part. Part I. Let $1_{\left\{\cdot \right\}}$ be an indicator variable. It is equal to one when the expression in {·} is true; otherwise, it is equal to zero. For any given i≠j, define

$$\xi_{ij}=\sum_{k=0,k\neq i,j}^{n}1_{\left\{t_{ik}>0,t_{jk}>0\right\}}.$$

Note that $\xi_{ij}$ is the sum of n−1 independent Bernoulli random variables, and for three distinct indices i,j,k,

$$E\xi_{ij}=P\left(t_{ik}>0\right)P\left(t_{jk}>0\right)={\left(1-{\left(1-p_{n}\right)}^{T}\right)}^{2}:=\eta_{n}.$$

Based on the Chernoff bound in [29], we have

$$\begin{array}{c} P\left(\xi_{ij}\leq \frac{1}{2}\left(n-1\right)\eta_{n}\right)\leq \exp\left(-\frac{1}{8}\left(n-1\right)\eta_{n}\right). \end{array}$$

It follows that

$$\begin{array}{c} P(\min_{i,j}\xi_{ij}\leq \frac{1}{2}\left(n-1\right)\eta_{n})\leq \sum_{i,j}P\left(\xi_{ij}\leq \frac{1}{2}\left(n-1\right)\eta_{n}\right)\leq \frac{(n+1)n}{2}\exp\left(-\frac{1}{8}\left(n-1\right)\eta_{n}\right). \end{array}$$

Since T≥1,

$$\eta_{n}={\left(1-{\left(1-p_{n}\right)}^{T}\right)}^{2}\geq {\left(1-\left(1-p_{n}\right)\right)}^{2}=p_{n}^{2}.$$

That is, with a probability of at least $1-\frac{(n+1)n}{2}\exp\left(-\frac{1}{8}\left(n-1\right)\eta_{n}\right)$, we have

(A12) $$\min_{i,j}\xi_{ij}\geq \frac{1}{2}\left(n-1\right)\eta_{n}\geq \frac{1}{2}\left(n-1\right)p_{n}^{2}.$$

Part II. For convenience, we introduce a non-negative array ${\left\{q_{ij}\right\}}_{i,j=1}^{n}$, where

$$q_{ij}:=-v_{ij},\;i\neq j;\;\;q_{ii}:=\sum_{k=1}^{n}v_{ik}=-v_{i0},\;\;i,j=1,\ldots ,n.$$

Let

$$\begin{array}{ccc} m:=\min_{\left(i,j\right)\in \{\left(i,j\right):q_{ij}>0\}}q_{ij}, & & M:=\max_{i,j}q_{ij},\\ t_{\max}:=\max_{i}t_{i}, & & t_{\min}:=\min_{i}t_{i}. \end{array}$$

It is clear that M≥m>0 and

$$q_{ij}\geq 0,\;\;q_{ij}=q_{ji},\;\;v_{ij}=-q_{ij},\;i\neq j,\;\;Mt_{\max}\geq v_{ii}=\sum_{k=1}^{n}q_{ik}\geq mt_{\min}.$$

Notice that

$$V^{-1}-S=\left(V^{-1}-S\right)\left(I-VS\right)+S\left(I-VS\right),$$

where *I* is a n×n identity matrix. Let X=I−VS, Y=SX and $Z=V^{-1}-S$; we have the recursion formula

Z=ZX+Y.

The goal is to give an upper bound of all $|z_{ij}|$. According the definitions of *S*, *V* and *X*, we have

$$\begin{array}{ccc} x_{ij} & = & \delta_{ij}-\sum_{k=1}^{n}v_{ik}s_{kj}\\ & = & \delta_{ij}-\sum_{k=1}^{n}v_{ik}\left(\frac{\delta kj}{v_{jj}}+\frac{1}{v_{00}}\right)\\ & = & \delta_{ij}-\frac{v_{ij}}{v_{ii}}-\frac{q_{ii}}{v_{00}}\\ & = & \left(1-\delta_{ij}\right)\frac{q_{ij}}{v_{jj}}-\frac{q_{ii}}{v_{00}}. \end{array}$$

and

$$\begin{array}{ccc} y_{ij} & = & \sum_{k=1}^{n}s_{ik}x_{kj}=\sum_{k=1}^{n}\left(\frac{\delta_{ik}}{v_{ii}}+\frac{1}{v_{00}}\right)\left(\left(1-\delta_{kj}\right)\frac{q_{kj}}{v_{jj}}-\frac{q_{kk}}{v_{00}}\right)\\ & = & \sum_{k=1}^{n}\frac{\delta_{ik}}{v_{ii}}\left\{\left(1-\delta_{kj}\right)\frac{q_{kj}}{v_{jj}}-\frac{q_{kk}}{v_{00}}\right\}+\sum_{k=1}^{n}\frac{1}{v_{00}}\left\{\left(1-\delta_{kj}\right)\frac{q_{kj}}{v_{jj}}-\frac{q_{kk}}{v_{00}}\right\}\\ & = & \frac{\left(1-\delta_{ij}\right)q_{ij}}{v_{ii}v_{jj}}-\frac{q_{ii}}{v_{ii}v_{00}}-\frac{q_{jj}}{v_{jj}v_{00}}. \end{array}$$

Since

$$0\leq \frac{q_{ij}}{v_{ii}v_{jj}}\leq \frac{M}{m^{2}t_{\min}^{2}},\;\;0\leq \frac{q_{ij}}{v_{ii}v_{00}}\leq \frac{M}{m^{2}t_{\min}^{2}},$$

for any different i,j,k, we have

(A13) $$|y_{ij}|\leq a:=\frac{2M}{m^{2}t_{\min}^{2}},\;\;\left|y_{ij}-y_{ik}\right|\leq a.$$

In view of the expressions of $x_{ij}$ and $y_{ij}$, we have

(A14) $$z_{ij}=\sum_{k=1}^{n}z_{ik}\left(1-\delta_{kj}\right)\frac{q_{kj}}{v_{jj}}-\sum_{k=1}^{n}z_{ik}\frac{q_{kk}}{v_{00}}+y_{ij},\;\;i,j=1,\ldots ,n.$$

Now, we fix an arbitrary *i* value and consider the upper bound of $\max_{j}\left|z_{ij}\right|$. Let α and β be such that

$$z_{i\alpha }=\max_{k=1,\ldots ,n}z_{ik},\;\;z_{i\beta }=\min_{k=1,\ldots ,n}z_{ik}.$$

Without loss of generality, we assume $z_{i\alpha }\geq \left|z_{i\beta }\right|$ (otherwise, we can invert the sign of $z_{ik}$ and repeat the same process). Below, we will show $z_{i\beta }\leq 0$. Note that this conclusion is not investigated in [20]. By multiplying $v_{jj}$ by both sides of (A14), we have

$$v_{jj}z_{ij}=\sum_{k=1}^{n}z_{ik}\left(1-\delta_{kj}\right)q_{kj}-\sum_{k=1}^{n}z_{ik}\frac{q_{kk}v_{jj}}{v_{00}}+v_{jj}y_{ij}.$$

Summarizing the above equations with j=1,…,n, we have

$$\sum_{j=1}^{n}v_{jj}z_{ij}=\sum_{k=1}^{n}\sum_{j=1}^{n}z_{ik}\left(1-\delta_{kj}\right)q_{kj}-\sum_{k=1}^{n}\sum_{j=1}^{n}z_{ik}\frac{q_{kk}v_{jj}}{v_{00}}+\sum_{j=1}^{n}v_{jj}\left(\frac{\left(1-\delta_{ij}\right)q_{ij}}{v_{ii}v_{jj}}-\frac{q_{ii}}{v_{ii}v_{00}}-\frac{q_{jj}}{v_{jj}v_{00}}\right).$$

Thus,

$$\begin{array}{ccc} & & \sum_{k=1}^{n}\sum_{j=1}^{n}z_{ik}\frac{q_{kk}v_{jj}}{v_{00}}+\sum_{k=1}^{n}z_{ik}q_{kk}\\ & = & \sum_{j=1}^{n}v_{jj}\left(\frac{\left(1-\delta_{ij}\right)q_{ij}}{v_{ii}v_{jj}}-\frac{q_{ii}}{v_{ii}v_{00}}-\frac{q_{jj}}{v_{jj}v_{00}}\right)\\ & = & \sum_{j=1}^{n}\frac{\left(1-\delta_{ij}\right)q_{ij}}{v_{ii}}-\frac{q_{ii}\sum_{j}v_{jj}}{v_{ii}v_{00}}-\sum_{j=1}^{n}\frac{q_{jj}}{v_{00}}\\ & = & -\frac{q_{ii}}{v_{ii}}-\frac{q_{ii}\sum_{j}v_{jj}}{v_{ii}v_{00}}=-\frac{q_{ii}}{v_{ii}v_{00}}\left(v_{00}+\sum_{j=1}^{n}v_{jj}\right) \end{array}$$

Thus,

$$\begin{array}{ccc} -\frac{q_{ii}}{v_{ii}v_{00}}\left(v_{00}+\sum_{j=1}^{n}v_{jj}\right) & = & \sum_{k=1}^{n}z_{ik}\frac{q_{kk}\sum_{j=1}^{n}v_{jj}}{v_{00}}+\sum_{k=1}^{n}z_{ik}q_{kk}\\ & \geq & z_{i\beta }\left(v_{00}+\sum_{j=1}^{n}v_{jj}\right), \end{array}$$

This shows

(A15) $$z_{i\beta }\leq -\frac{q_{ii}}{v_{ii}v_{00}}\leq 0.$$

Since $\sum_{k=1}^{n}q_{k\alpha }/v_{\alpha \alpha }=1$, we have

$$\sum_{k=1}^{n}z_{i\alpha }\frac{q_{k\alpha }}{v_{\alpha \alpha }}=\sum_{k=1}^{n}z_{ik}\left(1-\delta_{k\alpha }\right)\frac{q_{k\alpha }}{v_{\alpha \alpha }}-\sum_{k=1}^{n}z_{ik}\frac{q_{kk}}{v_{00}}+y_{i\alpha }.$$

In other words,

$$\sum_{k=1}^{n}\left[z_{i\alpha }-z_{ik}\left(1-\delta_{k\alpha }\right)\right]\frac{q_{k\alpha }}{v_{\alpha \alpha }}=-\sum_{k=1}^{n}z_{ik}\frac{q_{kk}}{v_{00}}+y_{i\alpha }.$$

Analogously, we have

$$\sum_{k=1}^{n}\left[z_{ik}\left(1-\delta_{k\beta }\right)-z_{i\beta }\right]\frac{q_{k\beta }}{v_{\beta \beta }}=\sum_{k=1}^{n}z_{ik}\frac{q_{kk}}{v_{00}}-y_{i\beta }.$$

Therefore,

(A16) $$\begin{array}{ccc} y_{i\alpha }-y_{i\beta } & = & \sum_{k=1}^{n}\left\{\left[z_{i\alpha }-z_{ik}\left(1-\delta_{k\alpha }\right)\right]\frac{q_{k\alpha }}{v_{\alpha \alpha }}+\left[z_{ik}\left(1-\delta_{k\beta }\right)-z_{i\beta }\right]\frac{q_{k\beta }}{v_{\beta \beta }}\right\}\\ & \geq & \left[\sum_{\left\{k:t_{\alpha k}>0,t_{k\beta }>0\right\}}\left(z_{i\alpha }-z_{i\beta }\right)\right]\times \frac{m}{Mt_{\max}}. \end{array}$$

The following calculations are based on the event $E_{n}$:

$$\left\{\min_{i\neq j}\xi_{ij}\geq \frac{1}{2}\left(n-1\right)p_{n}^{2},\;\;t_{\min}\geq \frac{1}{2}nTp_{n},\;\;t_{\max}\leq \frac{3}{2}nTp_{n}\right\}$$

In view of (A15) and (A16), we have

(A17) $$\begin{array}{ccc} z_{i\alpha } & \leq & z_{i\alpha }-z_{i\beta }\leq \frac{Mt_{\max}}{m}\times {\left[\min_{i\neq j}\xi_{ij}\right]}^{-1}\times \frac{2M}{m^{2}t_{\min}^{2}}\\ & \leq & \frac{M\cdot \frac{3}{2}nTp_{n}}{m}\times \frac{1}{\frac{1}{2}\left(n-1\right)p_{n}^{2}}\times \frac{2M}{m^{2}{\left(\frac{1}{2}nTp_{n}\right)}^{2}}\\ & = & \frac{24M^{2}}{n\left(n-1\right)m^{3}Tp_{n}^{3}} \end{array}$$

Note that $M=Tb_{n1}$ and $m=b_{n0}$. Based on Lemma 6 in the main text, we have

$$P\left(t_{\min}\geq \frac{1}{2}nTp_{n},t_{\max}\leq \frac{3}{2}nTp_{n}\right)\geq 1-2\left(n+1\right)\exp\left(-\frac{1}{8}nTp_{n}\right).$$

In view of inequality (A12), we have

$$P\left(E_{n}\right)\geq 1-\frac{(n+1)n}{2}e^{-\frac{1}{8}\left(n-1\right)p_{n}}-2\left(n+1\right)e^{-\frac{1}{8}nTp_{n}}.$$

If $p_{n}\geq 24\log n/n$, then

$$\frac{(n+1)n}{2}e^{-\frac{1}{8}\left(n-1\right)p_{n}}\leq O\left(\frac{1}{n}\right),\;\;\left(n+1\right)e^{-\frac{1}{10}np_{n}}=o\left(\frac{1}{n^{1.4}}\right),$$

such as

$$P\left(E_{n}\right)\geq 1-O\left(\frac{1}{n}\right).$$

Let $F_{n}$ be the event that $V^{-1}$ exists. Based on Lemma 1, if $p_{n}\geq 24\log n/n$, then

$$P\left(F_{n}\right)\geq 1-\exp\left(p_{n}T\left(n+1\right)\right)\geq 1-\frac{1}{n^{24}}.$$

Therefore,

$$P\left(E_{n}⋂F_{n}\right)\geq 1-O\left(\frac{1}{n}\right).$$

Substituting $M=Tb_{n1}$ and $m=b_{n0}$ into (A17) shows Lemma 2. □

### Appendix B.3. Proof of Lemma A3

**Proof of Lemma** **A3.** Recall that $\pi_{ij}=\beta_{i}-\beta_{j}$ and

$$H_{i}\left(\beta \right)=\sum_{j\neq i}t_{ij}\mu \left(\pi_{ij}\right)-a_{i},\;\;i=1,\ldots ,n.$$

The Jacobian matrix $H^{\prime }\left(\beta \right)$ of H(β) can be calculated as follows. By finding the partial derivative of $H_{i}$ with respect to β for i≠j, we have

$$\frac{\partial H_{i}\left(\beta \right)}{\partial \beta_{j}}=-t_{ij}\mu^{\prime }\left(\pi_{ij}\right),\;\;\frac{\partial H_{i}\left(\beta \right)}{\partial \beta_{i}}=\sum_{j\neq i}t_{ij}\mu^{\prime }\left(\pi_{ij}\right),$$

$$\frac{\partial^{2}H_{i}\left(\beta \right)}{\partial \beta_{i}\partial \beta_{j}}=-t_{ij}\mu^{\prime \prime }\left(\pi_{ij}\right),\;\;\frac{\partial^{2}H_{i}\left(\beta \right)}{\partial \beta_{i}^{2}}=\sum_{j\neq i}t_{ij}\mu^{\prime \prime }\left(\pi_{ij}\right).$$

When $\beta \in B(\beta^{*},\epsilon_{n})$, based on Condition (3c), we have

$$|\frac{\partial^{2}H_{i}\left(\beta \right)}{\partial \beta_{i}\partial \beta_{j}}|\leq b_{n2}t_{ij},\;\;i\neq j.$$

Let

$$g_{ij}\left(\beta \right)={\left(\frac{\partial^{2}H_{i}\left(\beta \right)}{\partial \beta_{1}\partial \beta_{j}},\ldots ,\frac{\partial^{2}H_{i}\left(\beta \right)}{\partial \beta_{n}\partial \beta_{j}}\right)}^{⊤}.$$

Therefore,

(A18) $$|\frac{\partial^{2}H_{i}\left(\beta \right)}{\partial \beta_{i}^{2}}|\leq t_{i}b_{n2},\;\;\left|\frac{\partial^{2}H_{i}\left(\beta \right)}{\partial \beta_{j}\partial \beta_{i}}\right|\leq b_{n2}t_{ij}.$$

This demonstrates that $∥g_{ii}{\left(\beta \right)∥}_{1}\leq 2t_{i}b_{n2}$. Note that when i≠j and k≠i,j,

$$\frac{\partial^{2}H_{i}\left(\beta \right)}{\partial \beta_{k}\partial \beta_{j}}=0.$$

Therefore, we have $∥g_{ij}{\left(\beta \right)∥}_{1}\leq 2t_{ij}b_{n2}$ for j≠i. Consequently, for vectors x,y⊂D, we have

$$\begin{array}{ccc} & & \max_{i=0,\ldots ,n}{∥H_{i}^{\prime }\left(x\right)-H_{i}^{\prime }\left(y\right)∥}_{1}\\ & \leq & \max_{i=0,\ldots ,n}\sum_{j=1}^{n}\left|\frac{\partial H_{i}\left(x\right)}{\partial x_{j}}-\frac{\partial H_{i}\left(y\right)}{\partial y_{j}}\right|\\ & = & \max_{i=0,\ldots ,n}\sum_{j=1}^{n}\left|\int_{0}^{1}{\left[g_{ij}\left(tx+\left(1-t\right)y\right)\right]}^{⊤}\left(x-y\right)dt\right|\\ & \leq & \max_{i=0,\ldots ,n}4b_{n2}t_{i}{∥x-y∥}_{\infty }\\ & = & 4b_{n2}t_{\max}{∥x-y∥}_{\infty }. \end{array}$$

This completes the proof. □

### Appendix B.4. Proof of Lemma A4

**Proof of Lemma** **A4.** Recall that $t_{i}=\sum_{j\neq i}t_{ij}$, and $a_{i}$ is the number of wins of subject *i* out of $t_{i}$ comparisons. Since all comparisons are mutually independent, $a_{i}$ is the sum of $m_{i}$ independent Bernoulli random variables given $t_{ij}=m_{ij}$ for j=0,…,n, where $m_{i}=\sum_{j\neq i}m_{ij}$. Based on [30]’s (1963) inequality, we have

$$\begin{array}{ccc} & & P\left(|a_{i}-E\left(a_{ij}|t_{ij},j=0,\ldots ,n\right)\left|\geq \sqrt{2m_{i}\log n}\right|t_{ij}=m_{ij},j=0,\ldots ,n\right)\\ & \leq & 2\exp\left\{-\frac{2m_{i}\log n}{m_{i}}\right\}=\frac{2}{n^{2}}. \end{array}$$

where $E(a_{ij}|t_{ij},j=0,\ldots ,n)$ is the conditional expectation given $t_{ij}$ for 0≤j≤n. Note that the upper bound of the above probability does not depend on $m_{ij}$. With the law of total probability, for fixed *i*,

$$\begin{array}{ccc} & & P\left(|a_{i}-\sum_{j}E\left(a_{ij}|t_{ij},j=0,\ldots ,n\right)\left|\geq \sqrt{2t_{i}\log n}\right|\right)\\ & = & \sum_{m_{i0}=0}^{T}\ldots \sum_{m_{in}=0}^{T}P\left(t_{ij}=m_{ij},j=0,\ldots ,n\right)\\ & & \times P\left(|a_{i}-\sum_{j}E\left(a_{ij}|t_{ij},j=0,\ldots ,n\right)\left|\geq \sqrt{2m_{i}\log n}\right|t_{ij}=m_{ij},j=0,\ldots ,n\right)\\ & \leq & 2n^{-2}\sum_{m_{i0}=0}^{T}\ldots \sum_{m_{in}=0}^{T}P\left(t_{ij}=m_{ij},j=0,\ldots ,n\right)\\ & \leq & 2\exp\left\{-\frac{2m_{i}\log n}{m_{i}}\right\}=\frac{2}{n^{2}}. \end{array}$$

Therefore,

$$\begin{array}{ccc} & & P\left(\max_{i=1,\ldots ,n}\left|a_{i}-\sum_{j}E\left(a_{ij}|t_{ij},j=0,\ldots ,n\right)\right|\geq \sqrt{2t_{\max}\log n}\right)\\ & \leq & P\left(\underset{i}{⋃}\left\{|a_{i}-Ea_{i}|\geq \sqrt{2t_{i}\log n}\right\}\right)\\ & \leq & \sum_{i=1}^{n}P\left(|a_{i}-Ea_{i}|\geq \sqrt{2t_{i}\log n}\right)\\ & \leq & n\times \frac{1}{n^{2}}=\frac{1}{n}. \end{array}$$

This completes the proof. □

### Appendix B.5. Proof of Lemma A5

**Proof of Lemma** **A5.** We first evaluate the uniform lower bound of $t_{i}$, i=0,…,n. Note that $t_{i}$ is the sum of nT independent and identically distributed (i.i.d.) binomial random variables, $Bin(T,p_{n})$. It can be also regarded as the sum of Tn, i.i.d., Bernoulli random variables. With the use of the Chernoff bound ([29]), we have

$$P\left(\min_{i=0,\ldots ,n}t_{i}<\frac{T}{2}np_{n}\right)\leq \sum_{i=0}^{n}P\left(t_{i}<\frac{T}{2}np_{n}\right)\leq \left(n+1\right)\exp\left(-\frac{T}{8}np_{n}\right).$$

Thus, with a probability of at least $1-\left(n+1\right)\exp\left(-\frac{T}{8}np_{n}\right)$,

$$\min_{i=0,\ldots ,n}t_{i}\geq \frac{T}{2}np_{n}.$$

Analogously, with the use of the Chernoff bound ([29]), we have

$$P\left(\max_{i=0,\ldots ,n}t_{i}>\frac{3}{2}nTp_{n}\right)\leq \sum_{i=0}^{n}P\left(t_{i}>\frac{3}{2}nTp_{n}\right)\leq \left(n+1\right)\exp\left(-\frac{1}{10}nTp_{n}\right),$$

and

$$P\left(\frac{1}{2}\sum_{i}t_{i}>\frac{3}{2}\left(n+1\right)nTp_{n}\right)\leq \exp\left(-\frac{1}{10}\left(n+1\right)nTp_{n}\right).$$

□

### Appendix B.6. Proof of Lemma A6

**Proof of Lemma** **A6.** Write

$$\begin{array}{c} H=H\left(\beta^{*}\right),\;\;V^{-1}=H^{\prime }\left(\beta^{*}\right),\\ E^{*}\left(\cdot \right)=E\left(\cdot |t_{ij},0\leq i,j\leq n\right),\;\;{\mathrm{Cov}}^{*}\left(\cdot \right)=\mathrm{Cov}\left(\cdot |t_{ij},0\leq i,j\leq n\right). \end{array}$$

Then,

$$H=E^{*}\left(a\right)-a.$$

Let $W=V^{-1}-S$. Note that $U={\mathrm{Cov}}^{*}\left(H\right)$. Via direct calculations, we have

$${\mathrm{Cov}}^{*}\left(WH\right)=\left(V^{-1}-S\right)U\left(V^{-1}-S\right):=I_{1}+I_{2}$$

where $I_{1}=V^{-1}-S+SVS-S$ and $I_{2}=\left(V^{-1}-S\right)\left(U-V\right)\left(V^{-1}-S\right)$. It is easy to verify

$${\left(SVS-S\right)}_{ij}=\frac{v_{i0}}{v_{ii}v_{00}}+\frac{v_{0j}}{v_{jj}v_{00}}-\frac{\left(1-\delta_{ij}\right)v_{ij}}{v_{ii}v_{jj}}.$$

Therefore,

$$\max_{i,j}\left|{\left(SVS-S\right)}_{ij}\right|\leq \frac{3b_{n1}}{t_{\min}^{2}b_{n0}^{2}}.$$

Based on Lemmas 3 and 5 in the main text, we have

$$I_{1}=O_{p}\left(\frac{b_{n1}^{2}}{n^{2}b_{n0}^{3}p_{n}^{5}}\right).$$

Now, we evaluate $I_{2}$. Direct calculations give

$$\begin{array}{cc} \; & \;\;{\left[\left(V^{-1}-S\right)\left(U-V\right)\left(V^{-1}-S\right)\right]}_{ij}\\ = & \;\;\sum_{k,s}{\left(V^{-1}-S\right)}_{ik}{\left(U-V\right)}_{ks}{\left(V^{-1}-S\right)}_{sj}\\ = & \;\;O\left({\left(\frac{b_{n1}^{2}}{n^{2}b_{n0}^{3}p_{n}\eta_{n}}\right)}^{2}\right)\sum_{k,s}\left|{\left(U-V\right)}_{ks}\right|\\ = & \;\;O\left({\left(\frac{b_{n1}^{2}}{n^{2}b_{n0}^{3}p_{n}\eta_{n}}\right)}^{2}\right)\times 2\left(b_{n1}+1/4\right)\sum_{i}t_{i}\\ = & \;\;O(\frac{b_{n1}^{5}}{n^{2}b_{n0}^{6}p_{n}^{5}}). \end{array}$$

where the second inequality is due to Lemma 3 and the third inequality is due to $p_{ij}\left(1-p_{ij}\right)\leq 1/4$ and $\mu_{ij}^{\prime }\left(\beta \right)\leq b_{n1}$. Therefore, we have

$$I_{1}+I_{2}=O_{p}\left(\frac{b_{n1}^{5}}{n^{2}b_{n0}^{6}p_{n}^{5}}\right).$$

□
